# Plasma Exosomal Proteomics Identifies Differentially Expressed Proteins as Biomarkers for Acute Myocardial Infarction

**DOI:** 10.3390/biom15040583

**Published:** 2025-04-15

**Authors:** Jie Zhou, Hai-Tao Hou, Huan-Xin Chen, Yu Song, Xiao-Lin Zhou, Li-Li Zhang, Hong-Mei Xue, Qin Yang, Guo-Wei He

**Affiliations:** 1 Department of Cardiac Surgery & The Institute of Cardiovascular Diseases, TEDA International Cardiovascular Hospital, Tianjin University, Tianjin 300457, China; zhoujie1999@tju.edu.cn (J.Z.); houht@tedaich.com (H.-T.H.); chenhx@tedaich.com (H.-X.C.); zhang1102_@tju.edu.cn (L.-L.Z.); xuehm@tedaich.com (H.-M.X.); yangq@tedaich.com (Q.Y.); 2 Tianjin Key Laboratory of Molecular Regulation of Cardiovascular Diseases and Translational Medicine, Tianjin 300457, China; 3 Department of Cardiac Surgery & The Institute of Cardiovascular Diseases, TEDA International Cardiovascular Hospital, Chinese Academy of Medical Sciences, Tianjin 300457, China; 4 The Institute of Cardiovascular Diseases & Critical Care Unit, Department of Cardiology, TEDA International Cardiovascular Hospital, Tianjin University & Chinese Academy of Medical Sciences, Tianjin 300457, China; songy@tedaich.com (Y.S.); zhouxl@tedaich.com (X.-L.Z.)

**Keywords:** acute coronary syndrome, STEMI, NSTEMI, biomarker, proteomics, exosomes

## Abstract

Myocardial infarction (MI), including ST-elevation MI (STEMI) and non-ST-elevation MI (NSTEMI), has been the leading cause of hospitalization and death. Exosomes participate in many physiological and pathological processes and have important effects on cell communication and function. This study analyzed the proteomic characteristics of plasma exosomes with the discovery of exosomal differentially expressed proteins (DEPs) in MI patients. Proteomics technology was used to identify the plasma exosomal DEPs in 41 patients in STEMI, NSTEMI, unstable angina, and CONTROL groups, and 406 exosomal DEPs were discovered. Further, 36 selected exosomal DEPs were validated with parallel reaction monitoring (PRM) in a new cohort of STEMI, NSTEMI, and CONTROL groups, and 7 were successfully verified. There were three (F13A1, TSPAN33, and YWHAZ) in the STEMI group and six (F13A1, TSPAN33, ITGA2B, GP9, GP5, and PPIA) in the NSTEMI group, and all were down-regulated compared to the CONTROL group with high sensitivity and specificity in MI that may be developed as biomarkers for MI and may become possible therapeutic targets for MI. Bioinformatics analysis revealed that these seven exosomal DEPs are of great significance in the molecular mechanism of MI. Therefore, the present study has provided insights to further explore the pathological mechanism and possible therapeutic targets in MI.

## 1. Introduction

Cardiovascular disease is the main cause of death worldwide, which is estimated to kill 17.9 million people every year [1]. Among the deaths caused by cardiovascular diseases, ischemic heart disease (IHD) is the leading cause of death, accounting for 16% of the total number of deaths in the world. In the past two decades, the number of deaths caused by IHD increased, exceeding 2 million in 2019, reaching 8.9 million [2,3]. Acute coronary syndrome (ACS) is the most typical type of IHD and is the primary reason for adult hospitalization [4]. In 2019, the number of ACS inpatients was 1,266,000, including 1,248,000 cases of myocardial infarction (MI) and 18,000 cases of unstable angina pectoris (UA) in the U.S.A [5]. MI can be divided into ST-segment elevation MI (STEMI) and non-ST-segment elevation MI (NSTEMI) according to the presence or absence of ST-segment elevation in ECG.

Exosomes are extracellular vesicles with phospholipid bilayer membrane structure, which are secreted by all mammalian cell types and exist in almost all biological fluids and tissues [6,7,8,9,10,11,12]. The diameter of exosomes is about 30–200 nanometers. The exosomes carry many contents, including nucleic acid, protein, lipid, and sugar [4,7,10,12,13,14,15,16,17], and are carriers of cell information exchange and important tools of cell-to-cell communication, participating in the regulation of many pathophysiological processes [8,11,13,17,18,19,20,21]. Studies on exosomes mainly focus on the therapeutic function of exosomes in the field of regenerative medicine, drug delivery as a carrier, and biomarkers for disease diagnosis and prognosis [11,17,19].

Studies on exosomes related to ACS have been focused on plasma exosome profiling in STEMI patients with and without out-of-hospital cardiac arrest compared with chronic coronary syndrome [8], and assessed the potential of the exosome Cyr61 as a diagnostic biomarker for ACS, explored the role of Cyr61 in vascular remodeling in vitro [4], and the protective and repair effects of pericardial fluid exocrine clusterin on cardiomyocytes after MI [15]. However, compared with the traditional analysis of many plasma soluble molecules, exosome-related proteins are not easy to degrade, specific, and contain all heterogeneous information [9,11]. On the basis that exosomes are excellent samples for investigations, there are no reports on the differences in exosomal proteins among STEMI, NSTEMI, and healthy individuals (CONTROL).

For patients with MI, especially patients with NSTEMI who have no typical ECG changes, the discovery of new potential clinical biomarkers at the exosome level may provide additional diagnostic value for accurate characterization. Furthermore, exploring the molecular mechanism of exosomal DEPs may help to reveal the molecular mechanisms and the role of exosomes in MI.

The aim of this study was to compare the exosomal protein profiling and differential proteins among STEMI, NSTEMI, and CONTROL in order to reveal the characteristics of the exosomal proteins and to identify possible biomarkers in MI.

## 2. Material and Methods

### 2.1. Experimental Design

The proteins of plasma exosomes were identified using label-free quantitative proteomic analysis to identify the DEPs. The selected DEPs were verified by the parallel reaction monitoring method. The experimental samples were collected prospectively from patients with ACS (STEMI, NSTEMI, and UA) and healthy individuals as controls. For the clinical data and sample information of the patients, statistical analysis and bioinformatics analysis were carried out. For more information, see the description in each section below.

### 2.2. Study Design and Population

This study was approved by the Institutional Review Board of TEDA International Cardiovascular Hospital, Tianjin, China (Approval No. [2020]-0528-3, 28 May 2020), and informed consent was obtained from all the patients included in the study. All procedures were in accordance with the ethical standards of the responsible committee on human experimentation (institutional and national) and with the Helsinki Declaration of 1975, as revised in 2000.

From February 2022 to April 2023, plasma samples were prospectively collected from patients diagnosed with STEMI, NSTEMI, and UA. Blood samples were collected for STEMI, NSTEMI, and UA patients from the Emergency Department. Blood samples of patients with negative coronary angiography were collected as CONTROL. A total of 41 patients (20 in the discovery phase and 21 in the verification phase) were included in this study by matching the most relevant clinical variables, such as age, sex, and race. Detailed inclusion and exclusion criteria of the samples are shown in Supplemental Table S1.

STEMI refers to designated MI in patients with chest discomfort or other ischemic symptoms who developed new ST-segment elevations in two contiguous leads or new bundle branch blocks with ischemia repolarization patterns as a STEMI. Patients presenting without ST-segment elevation were designated as having NSTEMI [22]. Patients without ST-segment elevation exerted symptoms suggestive of cardiac ischemia without elevated biomarker values can be diagnosed as having UA [22,23]. The inclusion and exclusion criteria of STEMI, NSTEMI, and UA patients were according to the guidelines outlined by the 2017 European Society of Cardiology (ESC) [24]. The control subjects underwent conventional coronary angiography for atypical chest discomfort [25] that demonstrated normal coronary arteries as mentioned above.

**Figure 1:**
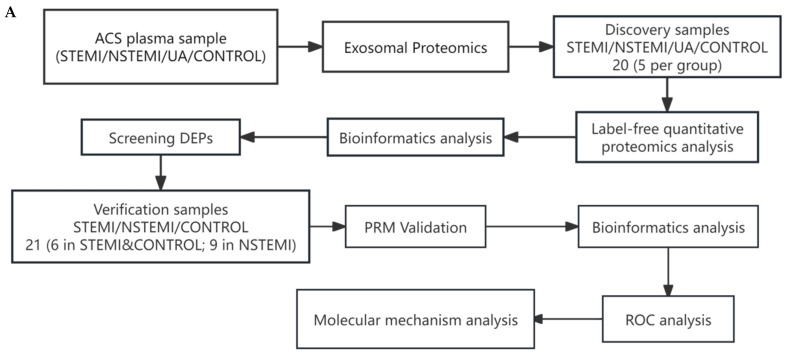

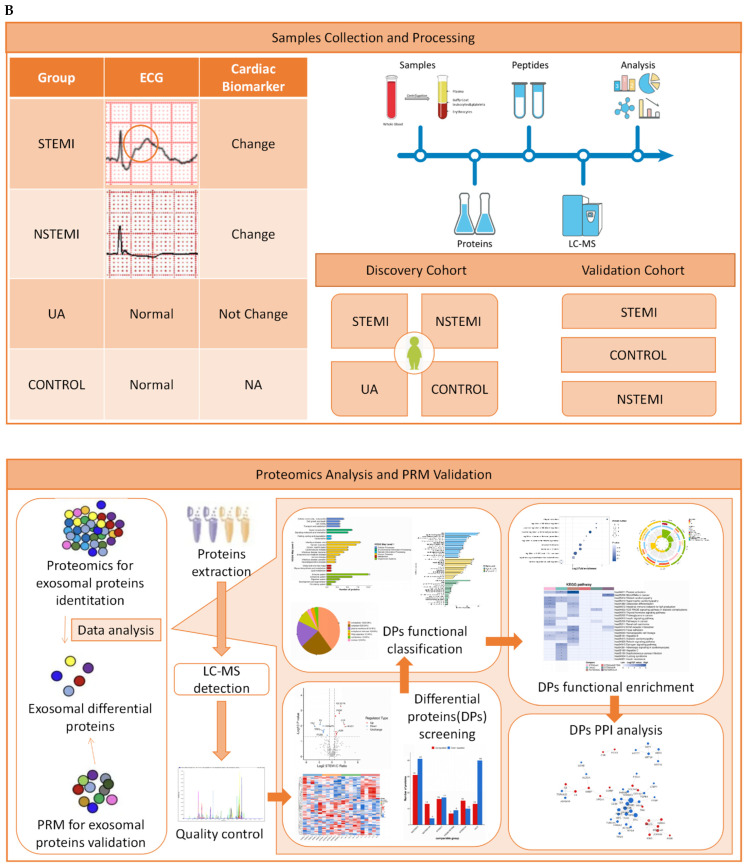
Brief flow chart (**A**) and workflow (**B**) for the proteomics study. Firstly, plasma samples were collected, and proteomics was used to identify exosomal differential proteins (DPs) in the STEMI, NSTEMI, UA, and CONTROL groups. After bioinformatics analysis and literature search, some of the DMs were selected and validated by PRM in the STEMI, NSTEMI, and CONTROL. The validation results were shown by grouping comparison charts and ROC curve charts in other figures.

The demographic and clinical data of the patients included in this study were taken from the patient’s medical records. The most relevant clinical variables, such as gender, age, and race, and possible clinical features, such as body mass index, risk factors, cardiac biomarkers (Myo, CKMB, hs-cTnI, BNP, D-Dimer), and laboratory biochemical test parameters, are shown in the Table 1. The experimental workflow is summarized and shown in Figure 1.

### 2.3. Samples Collection

Two mL blood samples were collected from the patient. The collected blood samples were centrifuged (1500× *g*, 10 min, 4 °C). The separated plasma and blood cells were stored in freezing tubes at −80 °C, respectively, until use.

### 2.4. Isolation and Identification of Exosomes

#### 2.4.1. Extraction and Purification of Exosomes

Plasma samples were used for exosome isolation with Umibio^®^ exosome isolation kits (Umibio, Cat. No: UR52136, Shanghai, China) according to the manufacturer’s instructions. The samples were thawed and centrifuged. Phosphate buffered saline (PBS) and blood PureExo solution were added to the obtained supernatant that was allowed to stand and centrifuge to keep the precipitate. The precipitate was resuspended with PBS and centrifuged, and the supernatant was retained to complete the extraction of exosomes. The harvested crude exosomes were purified by ExosomoePurafication Filter column and then centrifuged for subsequent experiments.

#### 2.4.2. Identification of Exosomes

Referring to the experience of published literature [26,27,28,29,30], we used transmission electron microscope (TEM) and Nanoparticle tracking analysis (NTA) to identify exosomes. The exosome particle size and concentration were measured by using NTA at Umibio (Shanghai) Co. Ltd with NanoSight NS300 (Malvern Panalytical, Malvern, UK) and corresponding software NTA 3.4 Build 3.4.4. Isolated exosome samples were appropriately diluted using 1X PBS buffer (Biological Industries, Beit Haemek, Israel) to measure the particle size and concentration. As for the TEM detection, the exosomes were first fixed on the sample-loaded copper net. The negative staining and detection were carried out.

### 2.5. Proteomics Analysis and Validation

#### 2.5.1. Identification of Exosomal Differentially Expressed Proteins (DEPs)

The plasma exosomes were analyzed by label-free quantitative proteomics. Proteins were extracted, detected by LC-MS (Liquid Chromatograph Mass Spectrometer, ThermoFisher, Waltham, MA, USA), and analyzed. The exosomal DEPs between two biological groups were screened by fold change (FC), and *p* value. In this study, FC ≥ 1.5 or ≤ 0.67, and *p* < 0.05 were simultaneously satisfied. Cluster analysis, correlation analysis, enrichment analysis, and receiver operating characteristic (ROC) analysis, etc., were carried out on the exosomal DEPs in each comparison. The proteomics study had four groups, including STEMI, NSTEMI, UA, and CONTROL groups (*n* = 5 in each group).

#### 2.5.2. Validation of Exosomal DEPs

After the exosomal DEPs were identified from the proteomics analysis, selected DEPs were verified by Parallel Reaction Monitoring (PRM), in a new cohort of patients and controls. The PRM study had three groups including STEMI, NSTEMI, and CONTROL groups (*n* = 6 in STEMI and CONTROL, *n* = 9 in NSTEMI). PRM is an ion monitoring technique based on high-resolution and high-precision mass spectrometry. It is most suitable for quantification of multiple proteins in complex samples with an attomole-level detection. (https://www.creative-proteomics.com/services/parallel-reaction-monitoring-prm.htm (accessed on 9 January 2025)).

#### 2.5.3. Bioinformatics Analysis

Using the Uniport database, we annotated the identified proteins, so as to thoroughly understand the functional characteristics of different proteins. The exosomal DEPs were screened from the identified proteins. The functional classification statistics, enrichment analysis, and cluster analysis of Gene Ontology (GO) and Kyoto Encyclopedia of Genes and Genomes (KEGG) were carried out. The protein-protein interaction (PPI) network relationship was established to clearly show the interaction between proteins. Finally, the functional annotation of pathways was made through the KEGG PATHWAY database to determine the main biochemical and signal transduction pathways in which the exosomal DEPs were involved.

### 2.6. Statistical Analysis

For the baseline data of patients, the classified variables were displayed in numbers and summarized as percentages and compared between different groups by chi-square test or Fisher exact test. Continuous variables were expressed as median (95% CI) and inter quartile range (IQR). One-way ANOVA, Welch one-way ANOVA, or Kruskal–Wallis test were used for the overall analysis, and Tukey HSD, Games–Howell, or Dunn’s test were used for multiple comparisons when appropriate.

The correlation analysis of exosomal DEPs was carried out by calculating the Spearman correlation coefficient, *p* < 0.05 was considered to be statistically significant.

In this study, all charts (except Figure 1 and Figure 5) were statistically analyzed and visualized by SPSS software (v 26.0), PSORTb (v3.0), Python software (v 3.9), R software (v 4.2.1), and related software packages.

## 3. Results

### 3.1. Baseline Characteristics of Patients

The baseline characteristics of patients are shown in Table 1 and Supplemental Table S2. There were no significant differences in age, sex, body mass index, and risk factors (including hypertension, diabetes, dyslipidemia, and smoking) among STEMI, NSTEMI, UA, and CONTROL groups (*p* > 0.05). As for the current biomarkers used clinically, there were no significant differences among STEMI, NSTEMI, and UA in Myo, CKMB, hs-TnI, and D-Dimer (*p* > 0.05), while there was a significant difference in BNP between STEMI and NSTEMI (*p* = 0.015). The levels of AST, AST/ALT, TP, and UREA were changed significantly among the groups.

### 3.2. Characterization of Exosomes

The basic characteristics of exosomes were identified by TEM and NTA. As shown in Supplemental Figure S1A, plasma-derived exosomes were clearly observed in each sample. These particles were about 30–200 nanometers in size, and some exosomes were in a classic dish shape. In addition, NTA results showed that all the samples presented good uniformity, and most of the particles were distributed in the particle size range of 110–170 nm (Supplemental Figure S1B). These results showed that the exosomes derived from patients’ plasma have good quality and purity and were suitable for the subsequent proteomics analysis.

### 3.3. Label-Free Quantitative Proteomics Profiling of Plasma Exosomes from Discovery Phase

#### 3.3.1. Screening of Exosomal DEPs

**Figure 2:**
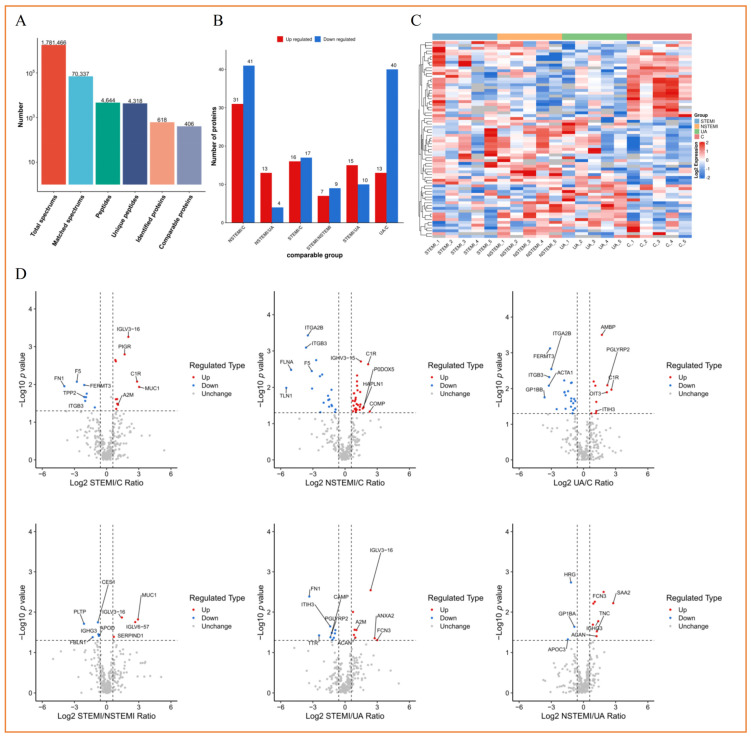

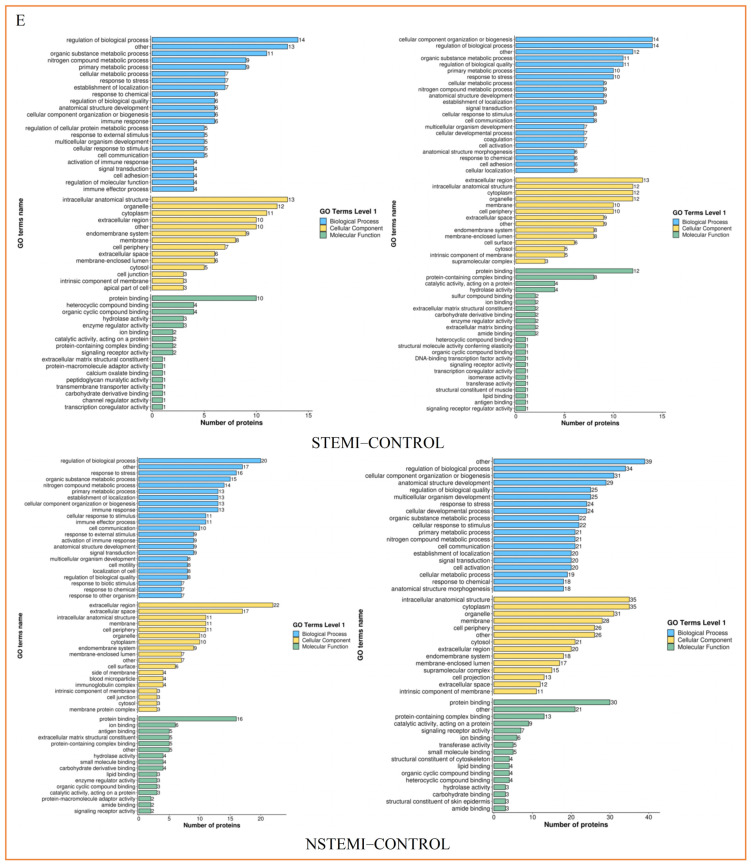

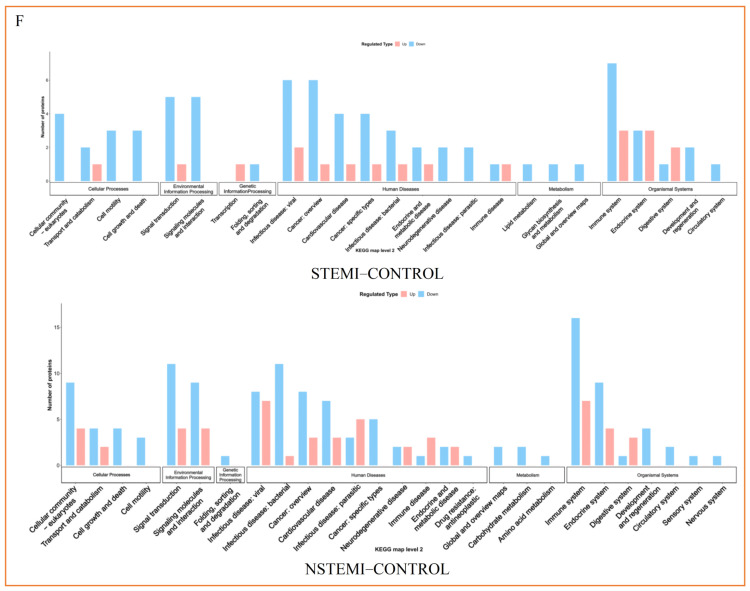
Screening and functional classification of exosomal differentially expressed proteins (DEPs): (**A**) proteins identification bar chart; (**B**) quantitative statistical chart of DEPs; (**C**) heat map of DEPs; (**D**) volcanic map of DEPs; (**E**) up-regulating (left) and down-regulating (right) GO classification bar chart of DEPs; (**F**) comparative bar chart of KEGG pathway classification of up-and down-regulated DEPs. [“C” means “CONTROL”].

In the discovery phase (STEMI, NSTEMI, UA, CONTROL), a total of 406 comparable proteins (Figure 2A) including 128 exosomal DEPs were identified. Among 128 DEPs, 33 DEPs (16 up, 17 down) between CONTROL and STEMI groups, 72 DEPs (31 up, 41 down) between CONTROL and NSTEMI groups, 52 DEPs (13 up, 40 down) between CONTROL and UA groups, 16 DEPs (7 up, 9 down) between STEMI and NTEMI groups, 25 DEPs (15 up, 10 down) between STEMI and UA groups, 17 DEPs (13 up, 4 down) between NSTEMI and UA groups were identified, respectively (Figure 2B). Figure 2C–F and Supplemental Figure S2A–C show the details of the exosomal DEPs. The up-regulated DEPs included PIGR, AMBP, C1R, and IGKC, and the down-regulated DEPs included F5, FERMT3, YWHAZ, ITGA2B, ITGB3, and F13A1 for the “STEMI-CONTROL” comparison and the “NSTEMI-CONTROL” comparison (Supplemental Figure S2A) or for all the comparisons (Supplemental Figure S2A) were identified. In addition, the heatmaps (Figure 2C and Supplemental Figure S2B) and clustering KEGG enrichment analyses (Supplemental Figure S2C) are shown.

#### 3.3.2. Functional Classification of Exosomal DEPs

As can be seen from the rose diagram of subcellular classification, the DEPs were mainly located in extracellular and cytoplasm (Supplemental Figure S2D). According to the GO classification bar graph synthesis, for the “STEMI-CONTROL” comparison and the “NSTEMI-CONTROL” comparison, the biological process (BP) that involved the most DEPs were “regulation of biological process” and “cellular component organization or biogenesis”. There were more DEPs distributed in the “intracellular anatomical structure” and “extracellular region” cellular component (CC), mainly involved in the “protein binding” and “protein-containing complex binding” related molecular function (MF) (Figure 2D). More specific results and other comparison results are given in Supplemental Figure S2E and Supplemental Table S3. According to the KEGG pathway classification diagram in Figure 2E and Supplemental Figure S2F, the DEPs were mainly concentrated in the related pathways of human diseases and organic systems, cancer, infectious diseases, cardiovascular disease, the immune system, and the endocrine system.

#### 3.3.3. Functional Enrichment Analysis of Exosomal DEPs

**Figure 3:**
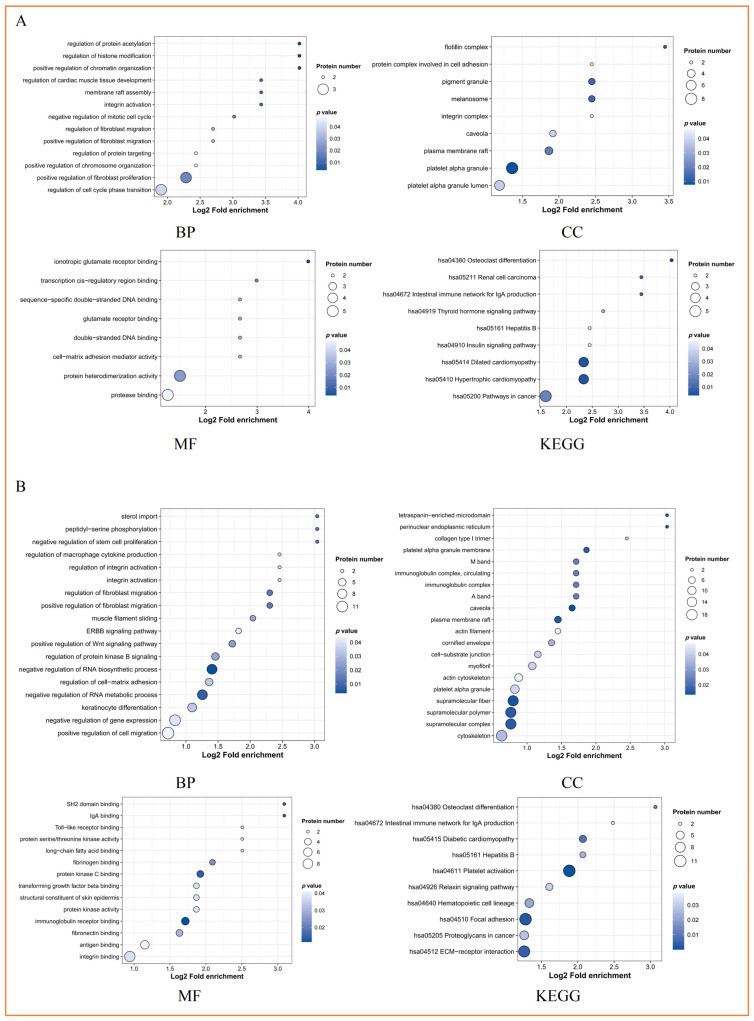

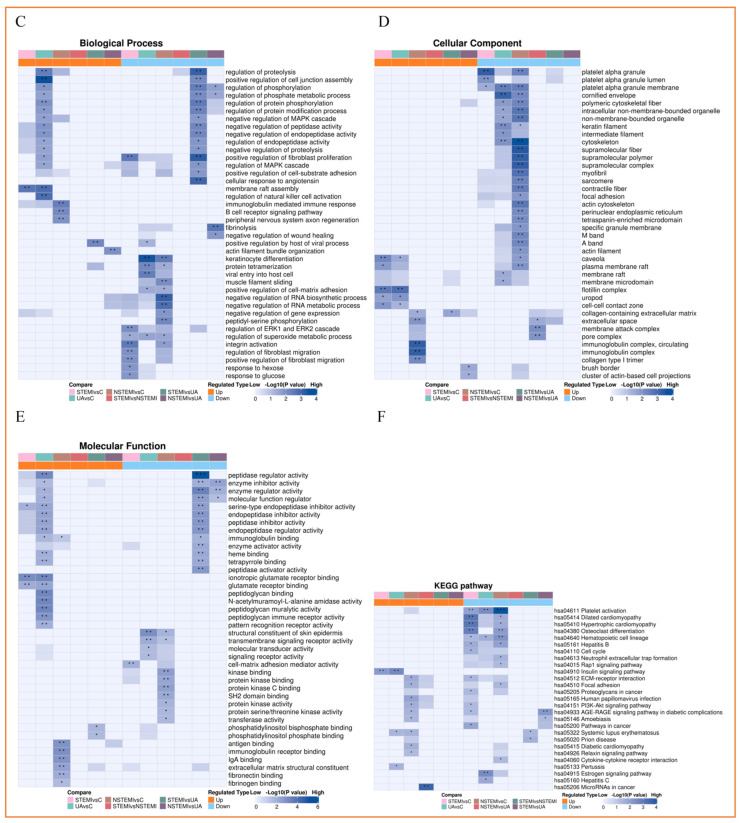

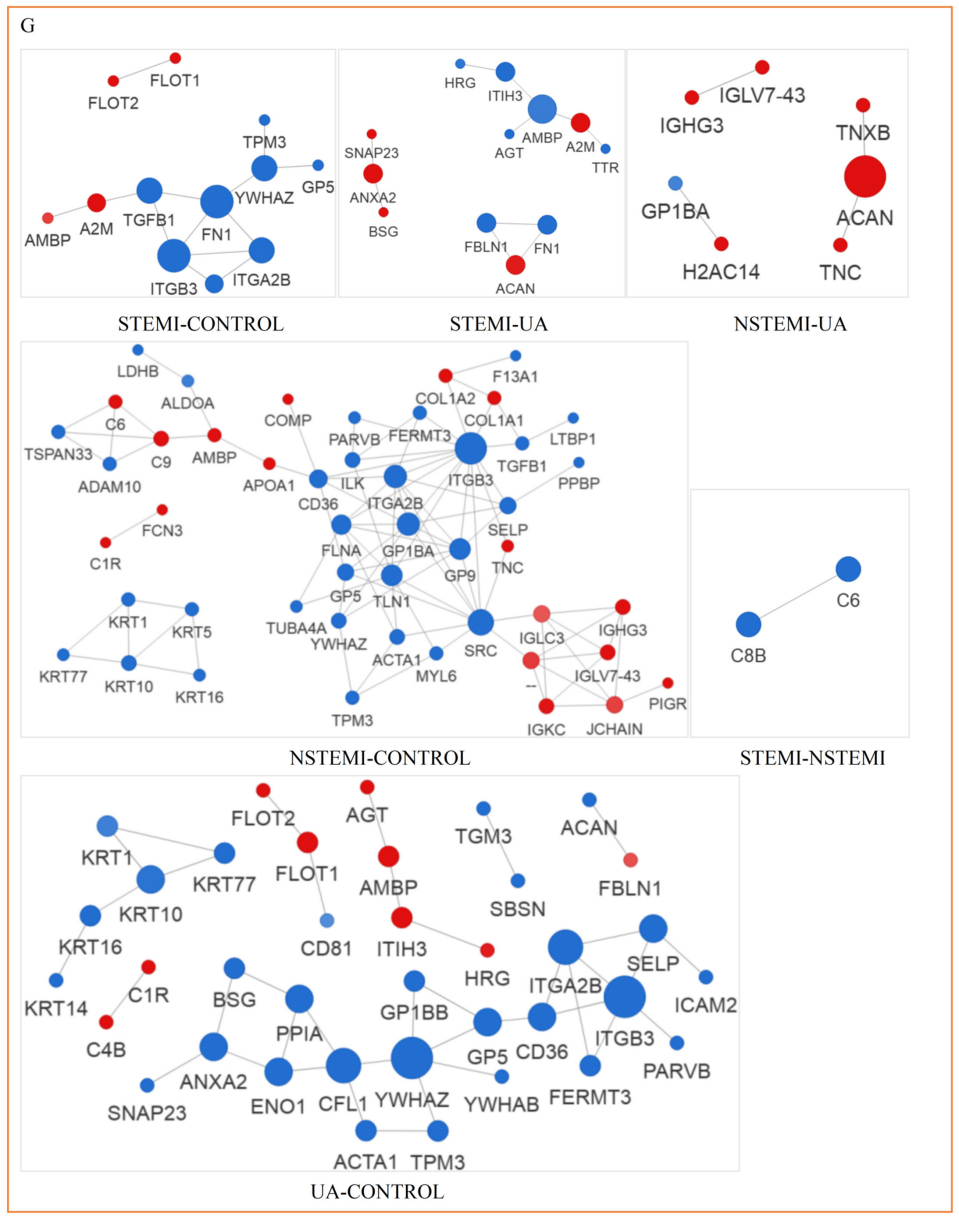
Functional enrichment analysis, cluster analysis, and protein-protein interaction (PPI) network analysis of exosomal DEPs: (**A**) bubble diagram of Gene Ontology (GO) and KEGG pathway enrichment analysis of DEPs in “STEMI-CONTROL”; (**B**) bubble diagram of Gene Ontology (GO) and KEGG pathway enrichment analysis of DEPs in “NSTEMI-CONTROL”; (**C**) cluster heat map of GO-BP; (**D**) cluster heat map of GO-CC; (**E**) cluster heat map of GO-MF; (**F**) cluster heat map of KEGG; (**G**) PPI analysis of DEPs. [“C” means “CONTROL”, “BP” means “biological process”, “CC” means “cell composition”, “MF” means “molecular function”. For C-F, * *p* < 0.05, ** *p* < 0.01, *** *p* < 0.001. For G, “red” means “up-regulated”, “blue” means “down-regulated”].

Figure 3A,B and Supplemental Figure S3 are GO enrichment analyses for BP, CC, MF, and KEGG pathway enrichment analysis of DEPs, respectively. Supplemental Table S4 summarizes the GO function and KEGG pathway with the most significant enrichment, the most enrichment, and the greatest enrichment degree of DEPs.

#### 3.3.4. Cluster Analysis of Exosomal DEPs

Figure 3C–F are the cluster heat maps of GO-BP, GO-CC, GO-MF, and KEGG, respectively. Supplemental Table S5 summarizes the number of GO function and KEGG pathways significantly clustered by DEPs in each comparison.

#### 3.3.5. Analysis of PPI Network of Exosomal DEPs

Figure 3G are PPI networks of different comparisons. The hinge proteins with the highest connectivity are ITGB3 and FN1 (STEMI-CONTROL), ITGB3, SRC, ITGA2B, GP1BA, and GP9 (NSTEMI-CONTROL), YWHAZ, and ITGB3 (UA-CONTROL).

### 3.4. PRM Analysis Revealed the Potential Biomarkers of STEMI and NSTEMI Patients in the Validation Phase

**Figure 4:**
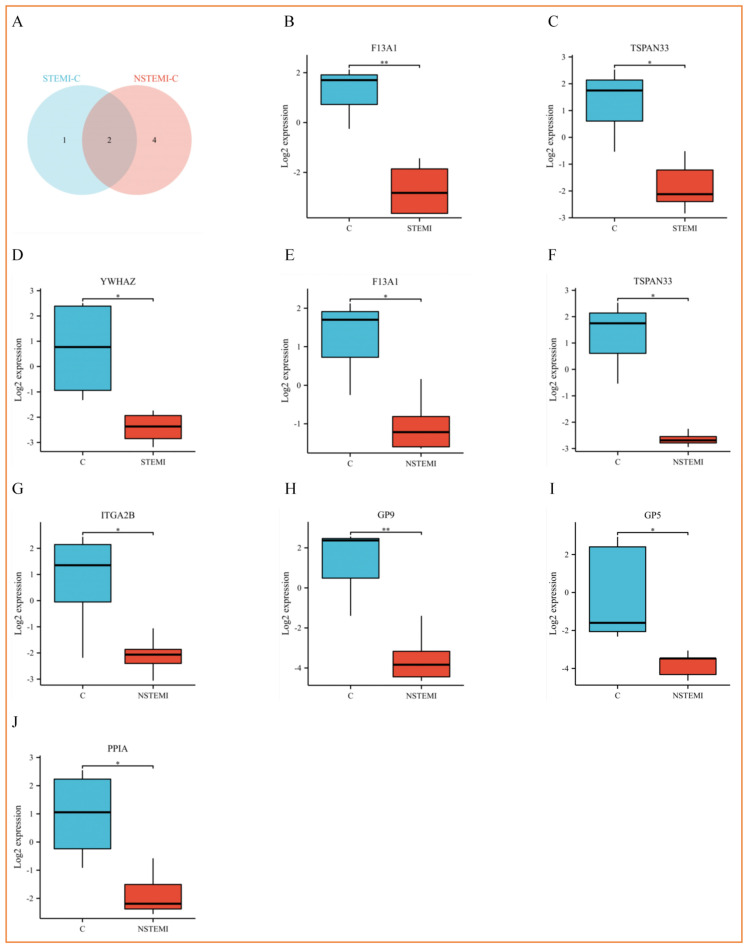

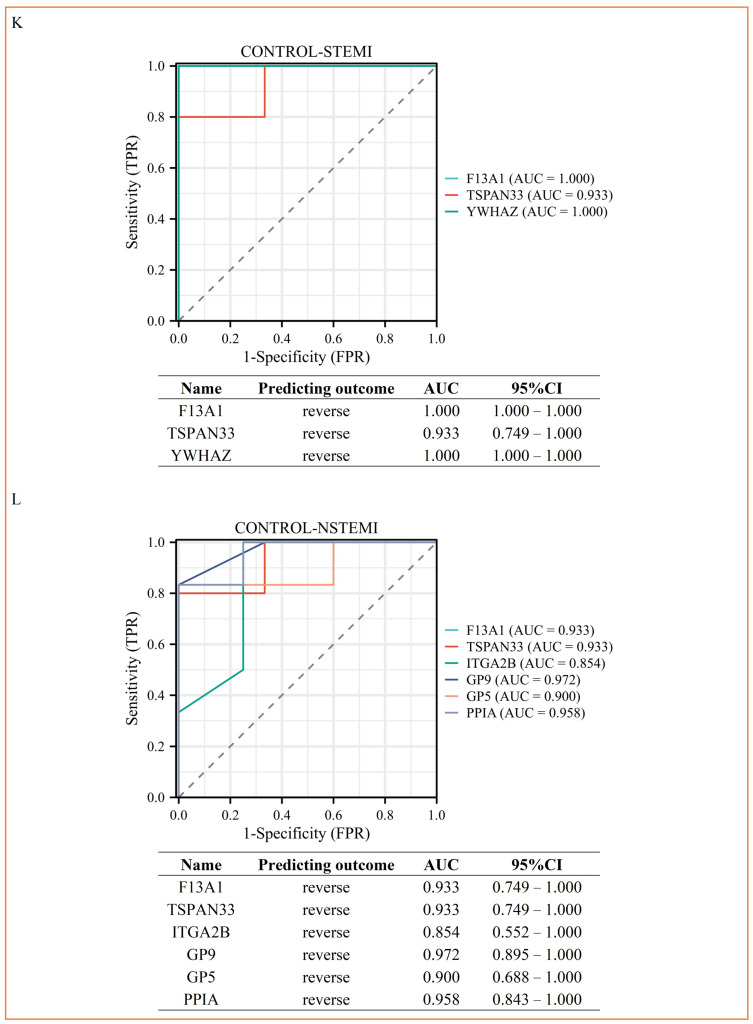
After the exosomal DEPs were identified from the proteomics analysis, selected 7 DEPs were verified by parallel reaction monitoring (PRM) in a new cohort of patients and controls. (**A**) Wayne diagram of DEPs. (**B**–**D**) Grouping comparison chart of DEPs expression in “STEMI-CONTROL”. (**E**–**J**) Grouping comparison chart of DEPs expression in “NSTEMI-CONTROL”. (For **B**–**J**, the error lines in the figure are mean ± standard error (SEM), respectively. * *p* < 0.05, ** *p* < 0.01.) (**K**) ROC curve chart of “STEMI-CONTROL”. (**L**) ROC curve chart of “NSTEMI-CONTROL”. (For (**K**) and (**L**), “reverse” stands for DEPs downward adjustment.) [“C” means “CONTROL”].

There were 36 exosomal DEPs selected from the discovery phase for further validation. Among these DEPs, 7 were successfully verified by matching the DEPs found in the discovery phase (Table 2, Figure 4A). Supplemental Figure S4 describes the ion peak area distribution of peptide fragments of DEPs.

Comparisons among the three groups are shown in Figure 4B–D. ROC analysis demonstrated that all AUC values were more than 0.8 (Figure 4K,L).

## 4. Discussion

### 4.1. The Key Findings

**Figure 5:**
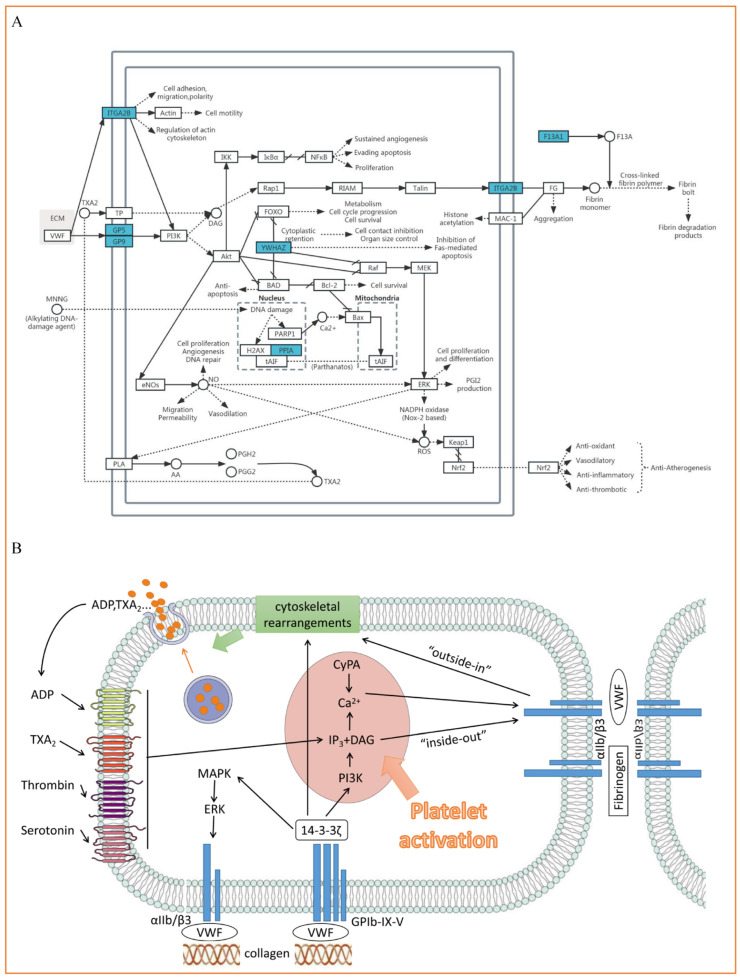
The KEGG pathway and mechanism of exosomal DEPs. (**A**) Diagram of integrated KEGG pathways of the 7 DEPs in MI discovered from this study. (The KEGG pathway integration map contains 6 DEPs, including 19 modified KEGG maps: “hsa04020”, “hsa04151”, “hsa04015”, “hsa04610”, “hsa00590”, “hsa00010”, “hsa04110”, “hsa04210”, “hsa04390”, “hsa04010”, “hsa05160”, “hsa04068”, “hsa04370”, “hsa05161”, “hsa04150”, “hsa04611”, “hsa04613”, “hsa05200”, “hsa05418”. Dots represent metabolites, boxes represent proteins, and blue is down-regulated. The line represents the pathway). (**B**) Schematic diagram of action mechanism of DEPs. (Collagen binds to platelet surface membrane receptor GPIb-IX-V through VWF and mediates intracellular signal transduction through 14-3-3ζ protein, which finally induces the increase in intracellular calcium concentration, induces platelet activation, and activates integrin αIIb/β3. Activated αIIb/β3 binds to fibrinogen or VWF, and platelet aggregation occurs. At the same time, this activation will also transmit signals to the cell, affect the changes in the cytoskeleton, control the release of soluble platelet agonists, and strengthen the activation of induced platelets). (VWF: von Willebrand factor) [Some of the pictures in Figure 5B are from smart.servier.com (accessed on 9 January 2025)].

In this exosomal proteomics analysis, we found that (1) during the first 2–8 h of chest pain attack, there were 7 exosomal DEPs in ACS patients; (2) there were 3 exosomal DEPs in STEMI and 6 exosomal DEPs in NSTEMI, which had high sensitivity and specificity; (3) these exosomal DEPs may be developed as biomarkers for diagnosis and potential targets for treatment of MI; and (4) the exosomal DEPs are included in several KEGG pathways (Figure 5A), which may reveal the mechanism of generation and action of DEPs found in this study.

The proteomics characteristics of plasma exosomes in patients with STEMI, NSTEMI, UA, and control group were analyzed in this study. Baseline characteristics showed that there were no significant differences in age, gender, BMI, and risk factors among the groups. The morphology and particle size (110–170 nm) of exosomes met the standard of exosome studies. A total of 128 DEPs were identified and enriched in cardiovascular diseases, immune system, and related pathways. PPI network analysis found key hub proteins such as ITGB3, ITGA2B, GP9, and YWHAZ. Finally, 36 DEPs were verified by PRM, and 7 of them showed good diagnostic potential (AUC > 0.8), suggesting a potential to be a biomarker of ACS. These 7 DEPs (shown in Table 2) are discussed in depth through the KEGG database and related literature as follows.

ITGA2B (αIIb), YWHAZ (14-3-3ζ), GP5 (V), and GP9 (IX) mediate platelet adhesion, activation, and aggregation through the synergistic effect of the Platelet activation pathway [31,32,33,34,35,36,37]. VWF, as a key bridge, connects the collagen matrix with platelet membrane receptor GPIb-IX-V complex and initiates downstream signal transduction, including the increase in Ca^2+^ concentration, PI3K activation, and conformational changes of integrin αIIb/β3, and finally promotes thrombosis [31,32,33,34,35,36,37]. CyPA (PPIA) participates in atherosclerosis and thrombosis through inflammatory and oxidative stress-related pathways (such as NF-κB and MAPK pathways) and regulates the dynamic balance of intracellular Ca^2+^ [38,39,40,41,42,43,44]. F13A1 (FXIII) plays a key role in the coagulation cascade, stabilizing thrombus by cross-linking fibrin and playing a protective role in heart repair after myocardial infarction [36,45,46,47,48,49,50]. TSPAN33 participates in inflammatory reactions through immunoregulation and cell adhesion-related pathways, but its specific mechanism in cardiovascular diseases needs further study [51,52,53,54,55]. These molecules interact with each other through complex signal networks to jointly regulate thrombosis, inflammatory reactions, and the development of cardiovascular diseases.

These findings provided new information for the understanding of the pathological changes in MI with regard to the proteins in the plasma exosome.

The details of the function of these 9 exosomal proteins (3 for STEMI and 6 for NSTEMI) can be found on the website https://www.uniprot.org/ (UniProt Consortium, accessed on 9 January 2025). The brief role of these proteins and their significance in MI are described below.

### 4.2. ITGA2B(αIIb), YWHAZ(14-3-3ζ), GP5(V) and GP9(IX) Participate in the Process of Platelet Adhesion, Activation and Aggregation

Von Willebrand factor (VWF) mediates the connection between collagen matrix and platelet membrane receptor glycoprotein GPIb-IX-V and locally produces soluble platelet agonists (mainly thrombin, adenosine diphosphate [ADP], and thromboxane A_2_ [TXA_2_]). With these effects, the initial activation and adhesion of platelets are initiated, and downstream signals are activated [31,32,33,34,35,36]. This signal reaction includes the increase in cytosolic Ca^2+^, the rearrangement of the cytoskeleton, the activation of PI3- kinase (PI3K), and the “inside-out” activation of integrin αIIb/β3. After activation, the conformation of αIIb/β3 changes, and through the combination of αIIb/β3 with fibrinogen or VWF, platelet adhesion and aggregation are mediated, and thrombosis is formed [31,32,34,35,36,37]. Subsequently, αIIb/β3 is activated to bind to ligands, and the “outside-in” signal transduction and the interaction with cytoskeleton proteins change, controlling post-adhesion events such as diffusion and contraction [36]. In cells, the GPIb-IX-V complex regulates platelet activation and adhesion under different physiological/pathological backgrounds by chelating 14-3-3ζ signal protein, regulating the binding state of receptor and VWF, and mediating intracellular signal transmission [31,34]. Activated platelets also release aggregation-promoting factors such as ADP, serotonin, and TXA_2_, and induce the production of secondary messenger diacylglycerol (DAG) and inositol 1,4,5-triphosphate (IP_3_), this process also leads to the increase in intracellular Ca^2+^ concentration and further induce platelet activation [31,33]. The increase in cytosolic calcium concentration will also regulate the release of ADP and TXA_2_; these molecules act as autocrine agonists to activate nearby platelets [31,37]. Platelets initially interact with VWF adsorbed on collagen through GPIb-IX-V, completing irreversible adhesion, and then soluble agonists TXA_2_ and ADP enhance αIIb/β3-dependent platelet aggregation [36,37]. Figure 5 describes the details of this process.

### 4.3. PPIA (Cypa) Is Involved in Inflammation and Thrombosis

Cyclophilin A (CyPA) is a ubiquitous chaperone protein with peptidyl-prolyl isomerase activity. This protein is distributed in the cytoplasm and extracellular space and mediates protein folding and signal transduction [38,39,40,41]. Previous studies have shown that CyPA is related to inflammation and oxidative stress, is a key pro-inflammatory mediator, and plays an important role in the pathogenesis of cardiovascular diseases [38,39,41,42,43]. CyPA is involved in various stages of atherosclerosis, such as activation of monocytes, injury formation, plaque rupture, and thrombosis [39]. Moreover, CyPA is also involved in Ca2+ regulation in cells (including platelets) and is released after platelet activation. This process is the key mechanism of arterial thrombosis [40,41,44]. The detailed explanation can be seen in Figure 5.

### 4.4. F13A1 (FXIII) Is Involved in Thrombosis and Causes Poor Prognosis

F13A1 is a subunit of factor XIII (FXIII). FXIII is a kind of blood-clotting protein and is the last enzyme activated in the coagulation cascade. Activated platelet aggregation accelerates the coagulation cascade reaction and can stabilize blood clots through cross-linking of fibrin molecules and αIIb/β3-dependent contraction [36,45]. FXIII in vivo is highly specialized for coagulation reactions and circulates in plasma as a potential complex with fibrinogen and thrombin [46]. At the initial stage of MI (0–20 min), FXIII participated in the formation of coronary thrombosis [47] and its level may decrease significantly. In addition, FXIII plays an important role in maintaining vascular permeability, angiogenesis, tissue remodeling, and promoting heart protection [46,47,48]. FXIII is a key molecule to promote the healing of cardiac wounds. After MI, the proliferation of fibroblasts controlled by FXIII and the fibrin network formed by FXIII are beneficial to the cardiovascular system and can rapidly reverse myocardial injury. It also prevents dilatation and rupture of the heart, heart failure, and cardiogenic shock caused by an enlarged infarction [49,50]. Therefore, a low level of FXIII is not conducive to cardiac prognosis. The detailed explanation can be seen in Figure 5B.

### 4.5. TSPAN33 (Tetraspanin-33)

Tetraspanins are a transmembrane protein superfamily involved in many physiological processes, including differentiation, adhesion, signal transduction, cell movement, and immune response [51]. TSPAN33 was first detected to be expressed in erythrocytes progenitor cells in mouse bone marrow with the effect of promoting erythropoiesis, indicating that it is related to hematopoiesis [52]. Studies have shown that TSPAN33 is expressed in B cells and affects cell adhesion, migration, endocytosis, plasma membrane regulation, and cytoskeleton dynamics, as well as immune response [53,54]. The expression of TSPAN33 is increased in some autoimmune diseases and lymphomas, and it also participates in the inflammatory process by regulating the expression of proinflammatory genes and the activation of different immune cells [51,54]. A study shows that TSPAN33 gene variation should be considered to affect β cell function and glucose metabolism [55]. However, there are no reports on the pathological role of TSPAN33 in cardiovascular diseases.

### 4.6. Influence of Cofounders of MI

In this study, the impact of the cofounders of MI should be considered. We have put great efforts into making the inclusion criteria for the MI patients to reduce any potential influence of the confounder such as age, sex, serious diseases of heart, lung, liver, brain, and kidney, and autoimmune diseases, etc. (Table S1). In addition, the influence of common cofounders for MI, such as hypertension, diabetes, dyslipidemia, and current smokers were statistically excluded (*p* > 0.05, Table 1). However, due to the sample size, the differences may not be shown statistically. Further, there were some cofounders such as AST, A/ALT, and TP that had differences among the groups. Nevertheless, the influence of the main cofounders as described above in this study may be minimal.

### 4.7. Study Limitations

In this study, the exosomal DEPs of patients with MI, including STEMI and NSTEMI, were identified for the first time. The study has limitations. First, as for any omics studies, the sample size is limited, even in the validation stage. Therefore, for the clinical use of the results from the omics study, further clinical trials with a larger cohort are necessary. Second, currently, the isolation of exosomes is technically difficult and would limit direct clinical translation. Third, the correlation between the DEPs in the exosomes and plasma and the correlation between the exosomes and the heart tissue at the infarcted area or its release to the blood is unknown. These questions cannot be answered by the present study. All the above formed a basis for future studies on the exosomes in MI.

## 5. Conclusions

This study analyzed the proteomic characteristics of plasma exosomes in patients with MI and revealed possible molecular mechanisms of exosomal DEPs in MI. There were seven DEPs with high sensitivity and specificity in MI that may be developed as biomarkers for MI and may become possible therapeutic targets for MI. Therefore, the present study has provided insights to further explore the pathological mechanism and possible therapeutic targets in MI.

## Figures and Tables

**Table 1:** Baseline characteristics for patients (patient numbers = 41).

	CONTROL	STEMI	NSTEMI	UA	*p*
	(*n* = 11)	(*n* = 11)	(*n* = 14)	(*n* = 5)	
Age, median (IQR)	62.00 (60.00–62.50)	62.00 (58.00–64.50)	64.50 (59.75–70.00)	62.00 (59.00–63.00)	0.147
Male, *n* (%)	6 (54.5%)	6 (54.5%)	7 (50%)	3 (60%)	0.983
BMI, kg/m^2^, median (IQR)	25.22 (23.22–28.17)	23.88 (23.56–27.25)	24.36 (23.27–25.33)	28.32 (27.73–28.41)	0.079
**Risk factors, *n* (%)**					
Hypertension, *n* (%)	7 (63.6%)	8 (72.7%)	9 (64.3%)	4 (80.0%)	0.889
Diabetes, *n* (%)	6 (54.5%)	3 (27.3%)	6 (42.9%)	4 (80.0%)	0.235
Dyslipidemia, *n* (%)	4 (36.4%)	2 (18.2%)	1 (7.1%)	1 (20.0%)	0.339
Current smoker, *n* (%)	4 (36.4%)	4 (36.4%)	3 (21.4%)	0 (0%)	0.382
**Cardiac Biomarkers, median (IQR)**					
Myo (ng/mL)	NA	423.00 (261.00–500.00)	267.00 (201.75–500.00)	113.00 (74.20–137.00)	0.055
CKMB (ng/mL)	NA	15.90 (2.65–59.35)	4.55 (3.03–9.60)	1.70 (1.10–2.10)	0.067
hs-cTnI (ng/mL)	NA	0.330 (0.115–4.330)	0.150 (0.050–1.435)	0.050 (0.050–0.050)	0.057
BNP (pg/mL)	NA	33.00 (21.50–55.80)	342.00 (96.55–811.00)	120.00 (10.30–123.00)	0.013
D-Dimer(ng/mL)	NA	240.0 (127.5–576.0)	463.0 (100.0–1389.2)	355.0 (207.0–480.0)	0.884
**Vessel lesion, *n* (%)**					
Single-vessel disease	0 (0%)	2 (18.2%)	0 (0%) *	1 (25.0%) *	0.301
Multi-vessel disease	0 (0%)	9 (81.8%)	9 (90.0%) *	3 (75.0%) *	0.760
**Laboratory data**					
Blood glucose (mmol/L)	6.05 (5.15–8.53)	8.60 (6.20–9.90)	9.85 (7.15–12.15)	7.40 (6.80–10.70)	0.113
ALT (U/L)	24.00 (19.25–28.50)	32.00 (19.50–59.00)	24.00 (14.50–39.50)	23.00 (23.00–35.00)	0.608
AST (U/L)	22.00 (19.25–23.00)	108.00 (43.00–197.00)	48.50 (24.25–86.25)	23.00 (17.00–37.00)	<0.001
AST/ALT	0.90 (0.80–1.00)	2.80 (2.50–3.80)	1.85 (1.23–3.18)	1.00 (1.00–1.10)	<0.001
GGT (U/L)	21.00 (16.00–26.75)	31.00 (17.00–40.00)	22.00 (13.75–34.50)	22.00 (18.00–27.00)	0.809
TP (g/L)	74.50 (68.50–75.75)	65.00 (63.50–68.50)	64.00 (59.50–66.50)	63.00 (62.00–69.00)	0.006
TBIL (μmol/L)	11.40 (8.45–12.93)	9.30 (6.40–12.95)	8.95 (7.83–13.73)	8.30 (5.00–13.90)	0.899
ALP (U/L)	73.00 (63.75–86.50)	87.00 (72.50–102.00)	74.50 (61.25–89.00)	74.00 (74.00–92.00)	0.567
Urea (mmol/L)	5.40 (4.60–5.95)	6.10 (5.65–7.45)	7.00 (5.40–10.38)	7.90 (6.10–9.50)	0.0498
Uric acid (μmol/L)	335.00 (247.50–351.5)	349.00 (311.00–376.00)	385.50 (312.25–454.5)	383.00 (349.00–437.00)	0.142
CREA (μmol/L)	60.00 (50.50–64.50)	60.00 (56.50–67.00)	83.50 (64.25–106.00)	65.00 (60.00–93.00)	0.075
Total cholesterol (mmol/L)	5.50 (3.98–6.38)	4.50 (3.85–4.90)	3.75 (3.20–4.68)	4.30 (4.10–5.60)	0.081
TG (mmol/L)	1.40 (1.02–1.78)	1.60 (1.22–1.86)	1.13 (0.76–1.88)	1.65 (1.46–3.67)	0.216
HDL-C (mmol/L)	1.16 (1.04–1.25)	1.00 (0.84–1.16)	0.92 (0.76–1.09)	0.80 (0.77–0.96)	0.125
LDL-C (mmol/L)	3.77 (2.31–4.49)	2.95 (2.14–3.30)	2.30 (1.89–2.80)	2.66 (2.35–2.98)	0.061
PLT(×10^9^/L)	242.00 (212.50–291.50)	242.00 (185.00–277.00)	207.00 (158.00–229.25)	247.00 (234.00–252.00)	0.292
**Length of stay (days)**	1.00 (1.00–2.00)	8.00 (6.50–9.00)	6.00 (4.25–7.75)	6.00 (6.00–13.00)	0.0494

* Some data not available. Data are expressed as median (IQR) or numbers (percentage). Abbreviation: BMI, body mass index; Myo, myoglobin; CKMB, creatine kinase myocardial band; hs-cTnI, high-sensitivity cardiac troponin I; BNP, B-type natriuretic peptide; ALT, alanine aminotransferase; AST, aspartate aminotransferase; GGT, gamma-glutamyl transferase; TP, total protein; TBIL, total bilirubin; ALP, alkaline phosphatase; CREA, creatinine; TG, triglyceride; HDL-C, high-density lipoprotein cholesterol; LDL-C, low-density lipoprotein cholesterol; PLT, platelet count.

**Table 2:** The specific information of 7 exosomal DEPs was successfully verified by PRM.

Comparison	Protein Name	Gene Name	Discovery Phase		Validation Phase	
			FC	*p* Value	FC(PRM/PRO)	*p* Value
**CONTROL-STEMI**	Coagulation factor XIII A chain	*F13A1*	0.466204068	0.040562299	0.07/0.47	8.05 × 10^−3^
	Tetraspanin-33	*TSPAN33*	0.001	0.001	0.1/0	1.25 × 10^−2^
	14-3-3 protein zeta/delta	*YWHAZ*	0.277126634	0.017480742	0.07/0.28	4.93 × 10^−2^
**CONTROL-NSTEMI**	Coagulation factor XIII A chain	*F13A1*	0.416860933	0.011631169	0.2/0.42	1.86 × 10^−2^
	Tetraspanin-33	*TSPAN33*	0.001	0.001	0.09/0	1.08 × 10^−2^
	Integrin alpha-IIb	*ITGA2B*	0.090198556	0.000373439	0.09/0.09	1.35 × 10^−2^
	Platelet glycoprotein IX	*GP9*	0.001	0.001	0.03/0	3.94 × 10^−3^
	Platelet glycoprotein V	*GP5*	0.001	0.001	0.05/0	2.22 × 10^−2^
	Peptidyl-prolyl cis-trans isomerase A	*PPIA*	0.33860234	0.022349546	0.11/0.34	3.68 × 10^−2^

## Data Availability

The data are available from the correspondence author with reasonable request.

## Supplementary Materials

The following supporting information can be downloaded at: https://www.mdpi.com/article/10.3390/biom15040583/s1, Figure S1: The identification of the plasma exosomes by electromicroscope (TEM) and nanoparticle tracking analysis (NTA) to show the characteristics; Figure S2: Screening and functional classification of exosomal differentially expressed proteins (DEPs); Figure S3: Functional enrichment analysis, cluster analysis, and protein-protein interaction (PPI) network analysis of exosomal DEPs; Figure S4: Ion peak area distribution of peptide fragments of exosomal DEPs; Table S1: Detailed inclusion and exclusion criteria of the study; Table S2: Multiple hypothesis test of factors with significant differences; Table S3: Summary of Top 3 GO function classification of exosomal DEPs; Table S4: Summary of the GO function and KEGG pathway enrichment analysis of exosomal DEPs; Table S5: Summary of the cluster number of GO function and KEGG pathways.
