# Supplementary material for: Plasma Exosomal Proteomics Identifies Differentially Expressed Proteins as Biomarkers for Acute Myocardial Infarction
Source: Biomolecules. 2025 Apr 15;15(4):583. doi: 10.3390/biom15040583 (PMC12025292; doi:10.3390/biom15040583)
Supplement: Supplementary file 1 [file biomolecules-15-00583-s001.zip › biomolecules-3457775-supplementary.pdf]

SUPPLEMENTAL FIGURE LEGENDS

Supplemental Figure S1 The identification of the plasma exosomes by electromicroscope (TEM) and nanoparticle tracking analysis (NTA) to show the characteristics.

Supplemental Figure S2 Screening and functional classification of exosomal differentially expressed proteins (DEPs). (A) Radar chart of DEPs. (B) Anova cluster analysis heat map of DEPs. The data points were divided into different clusters by using algorithms such as K-Means and hierarchical clustering to reveal the significance of the difference in different groups of data. (C) Anova KEGG enrichment analysis bubble diagram of DEPs. (D) Up-and down-regulation contrast rose diagram of subcellular classification of DEPs. (E) Regulated GO classification bar chart of DEPs. (F) Comparative bar chart of KEGG pathway classification of up-and down-regulated DEPs. [″C″ means ″CONTROL″]

Supplemental Figure S3 Functional enrichment analysis, cluster analysis, and protein-protein interaction (PPI) network analysis of exosomal DEPs. (A) Bubble diagram of Gene Ontology (GO) and KEGG pathway enrichment analysis of DEPs in ″UA-CONTROL″ . (B) Bubble diagram of Gene Ontology (GO) and KEGG pathway enrichment analysis of DEPs in ″STEMI-NSTEMI″ . (C) Bubble diagram of Gene Ontology (GO) and KEGG pathway enrichment analysis of DEPs in ″STEMI-UA″ . (D) Bubble diagram of Gene Ontology (GO) and KEGG pathway enrichment analysis of DEPs in ″NSTEMI-UA″ . [″C″ means ″CONTROL″]

Supplemental Figure S4 Ion peak area distribution of peptide fragments of exosomal DEPs.

Supplemental Figure S1

A

Group	Concentration(Particles/mL)	Size(nm)	TEM photograph
**CONTROL**			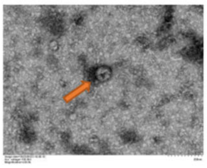
**STEMI**	10^7-10^12	30-200	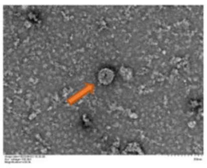
**NSTEMI**			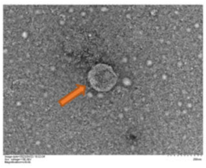

B

Group	Concentration(Particles/mL)	Size(nm)	NTA picture
**CONTROL**	6.40E+09	116.3	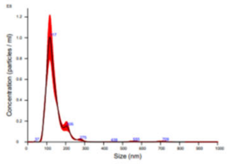
**STEMI**	3.15E+09	167.4	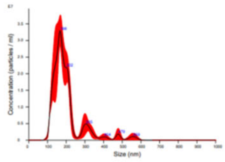
**NSTEMI**	1.19E+09	141.4	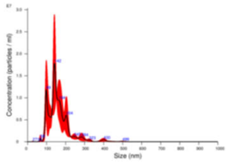

Supplemental Figure S2

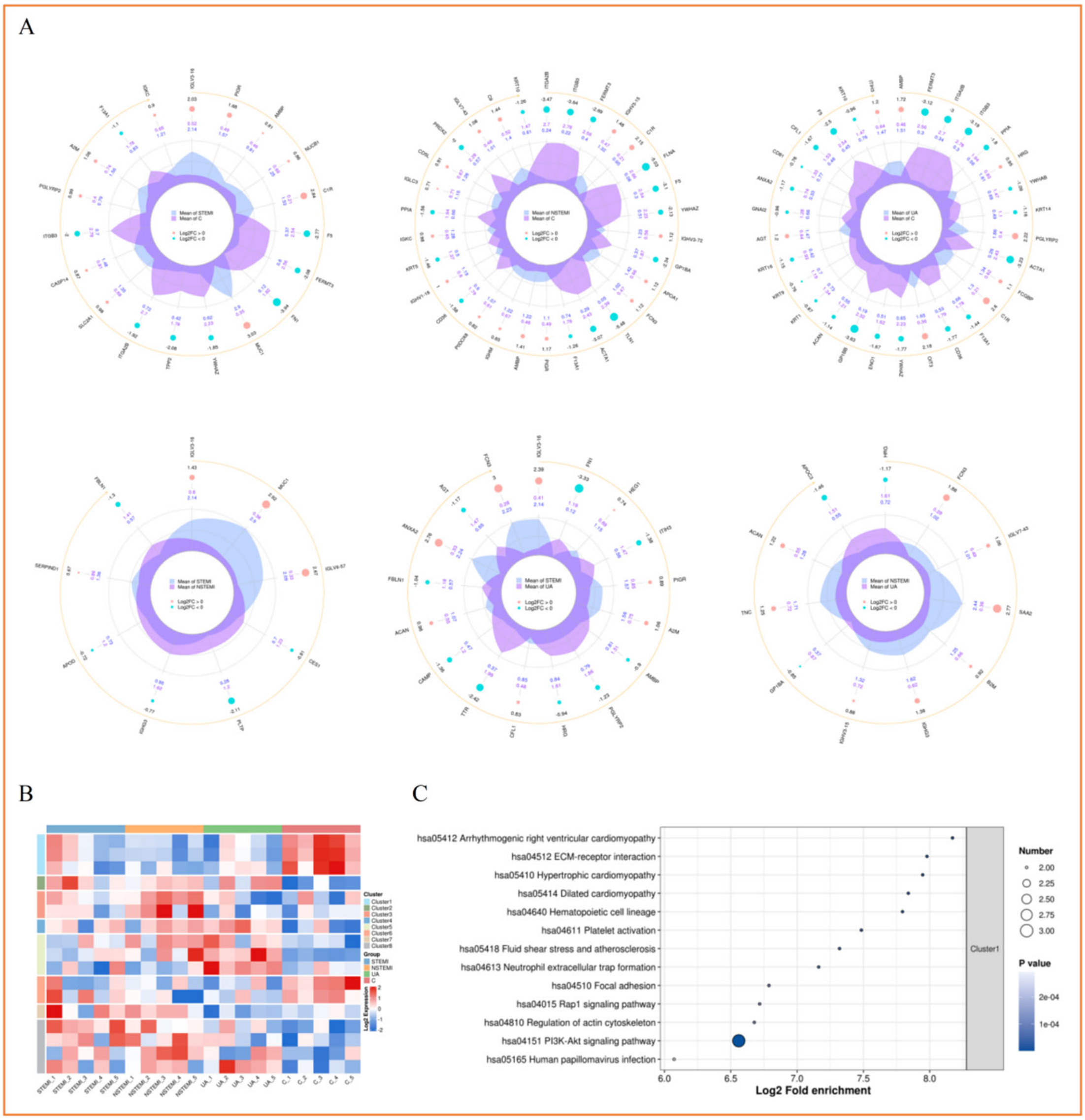

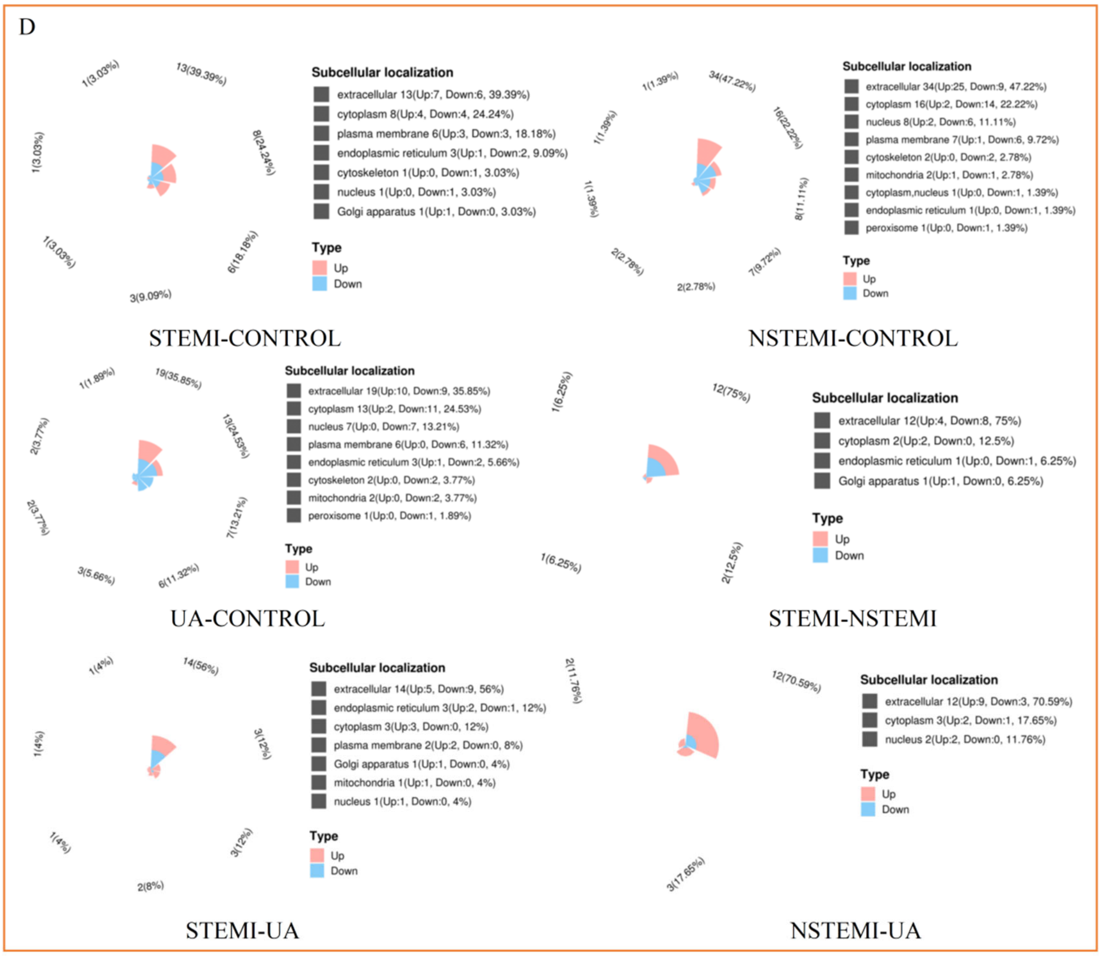
D
Subcellular localization
extracellular 13(Up:7, Down:6, 39.39%)
cytoplasm 8(Up:4, Down:4, 24.24%)
plasma membrane 6(Up:3, Down:3, 18.18%)
endoplasmic reticulum 3(Up:1, Down:2, 9.09%)
cytoskeleton 1(Up:0, Down:1, 3.03%)
nucleus 1(Up:0, Down:1, 3.03%)
Golgi apparatus 1(Up:1, Down:0, 3.03%)
Type
Up
Down
STEMI-CONTROL
Subcellular localization
extracellular 34(Up:25, Down:9, 47.22%)
cytoplasm 16(Up:2, Down:14, 22.22%)
nucleus 8(Up:2, Down:6, 11.11%)
plasma membrane 7(Up:1, Down:6, 9.72%)
cytoskeleton 2(Up:0, Down:2, 2.78%)
mitochondria 2(Up:1, Down:1, 2.78%)
cytoplasm,nucleus 1(Up:0, Down:1, 1.39%)
endoplasmic reticulum 1(Up:0, Down:1, 1.39%)
peroxisome 1(Up:0, Down:1, 1.39%)
Type
Up
Down
NSTEMI-CONTROL
Subcellular localization
extracellular 19(Up:10, Down:9, 35.85%)
cytoplasm 13(Up:2, Down:11, 24.53%)
nucleus 7(Up:0, Down:7, 13.21%)
plasma membrane 6(Up:0, Down:6, 11.32%)
endoplasmic reticulum 3(Up:1, Down:2, 5.66%)
cytoskeleton 2(Up:0, Down:2, 3.77%)
mitochondria 2(Up:0, Down:2, 3.77%)
peroxisome 1(Up:0, Down:1, 1.89%)
Type
Up
Down
UA-CONTROL
Subcellular localization
extracellular 12(Up:4, Down:8, 75%)
cytoplasm 2(Up:2, Down:0, 12.5%)
endoplasmic reticulum 1(Up:0, Down:1, 6.25%)
Golgi apparatus 1(Up:1, Down:0, 6.25%)
Type
Up
Down
STEMI-NSTEMI
Subcellular localization
extracellular 14(Up:5, Down:9, 56%)
endoplasmic reticulum 3(Up:2, Down:1, 12%)
cytoplasm 3(Up:3, Down:0, 12%)
plasma membrane 2(Up:2, Down:0, 8%)
Golgi apparatus 1(Up:1, Down:0, 4%)
mitochondria 1(Up:1, Down:0, 4%)
nucleus 1(Up:1, Down:0, 4%)
Type
Up
Down
STEMI-UA
Subcellular localization
extracellular 12(Up:9, Down:3, 70.59%)
cytoplasm 3(Up:2, Down:1, 17.65%)
nucleus 2(Up:2, Down:0, 11.76%)
Type
Up
Down
NSTEMI-UA

E

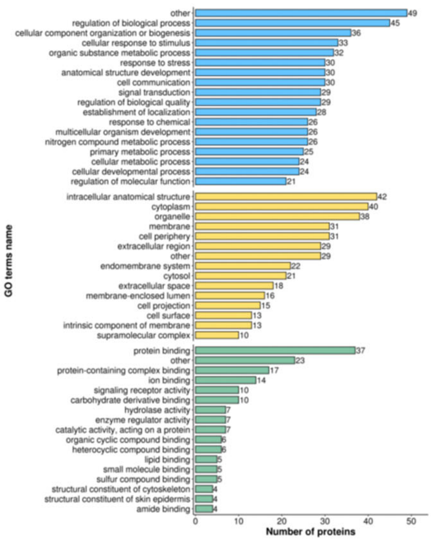

UA-CONTROL

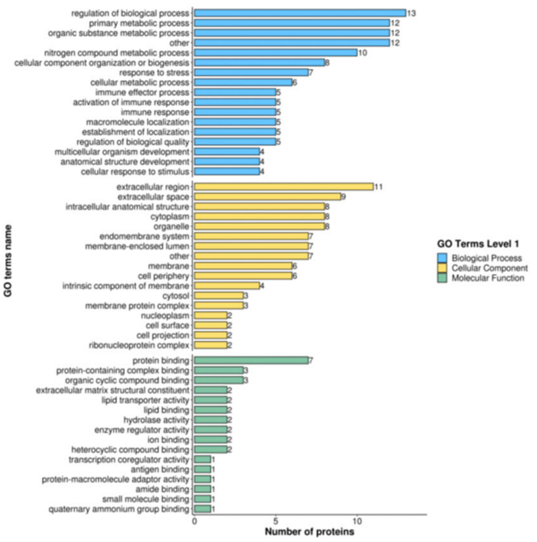

STEMI-NSTEMI

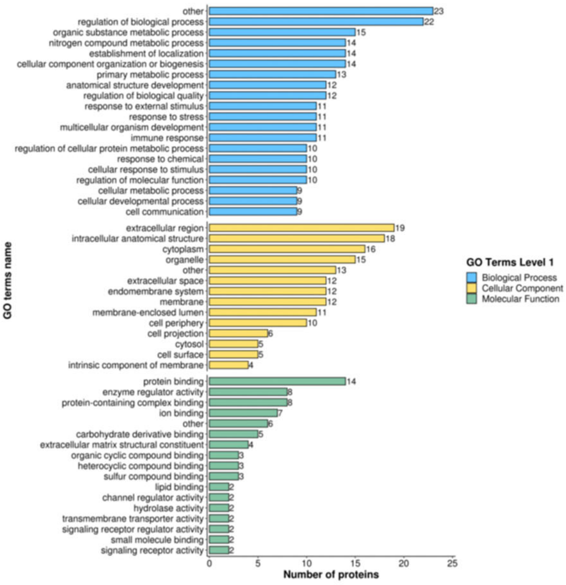

STEMI-UA

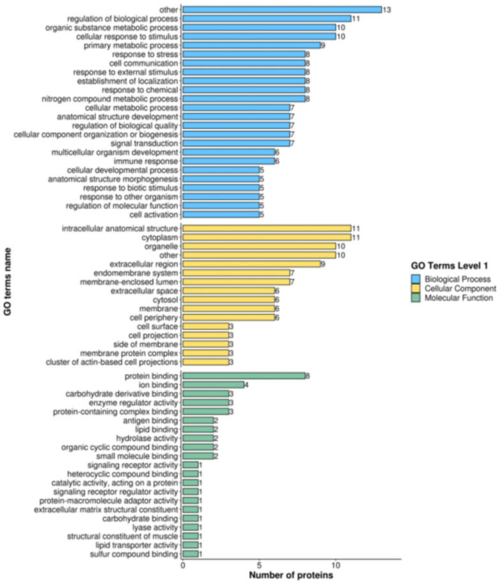

NSTEMI-UA

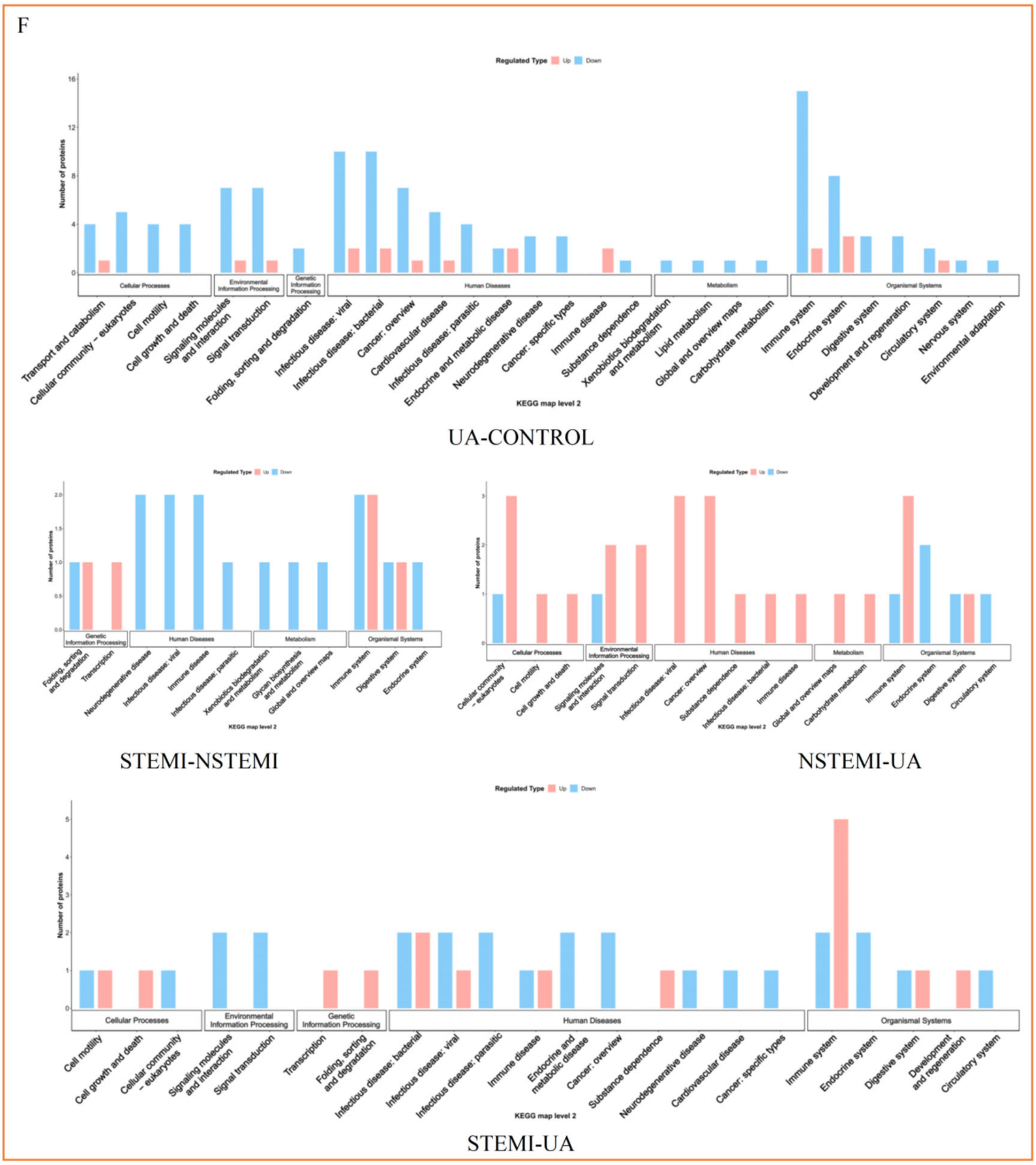

F
Regulated Type Up Down
Number of proteins
KEGG map level 2
Cellular Processes
Environmental Information Processing
Genetic Information Processing
Human Diseases
Metabolism
Organismal Systems
Transport and catabolism
Cellular community - eukaryotes
Cell motility
Cell growth and death
Signaling molecules and interaction
Signal transduction
Folding, sorting and degradation
Infectious disease: viral
Infectious disease: bacterial
Cancer: overview
Cardiovascular disease
Infectious disease: parasitic
Endocrine and metabolic disease
Neurodegenerative disease
Cancer: specific types
Immune disease
Substance dependence
Xenobiotics biodegradation and metabolism
Lipid metabolism
Global and overview maps
Carbohydrate metabolism
Immune system
Endocrine system
Digestive system
Development and regeneration
Circulatory system
Nervous system
Environmental adaptation
UA-CONTROL
Transcription
Glycan biosynthesis and metabolism
STEMI-NSTEMI
NSTEMI-UA
STEMI-UA

Supplemental Figure S3

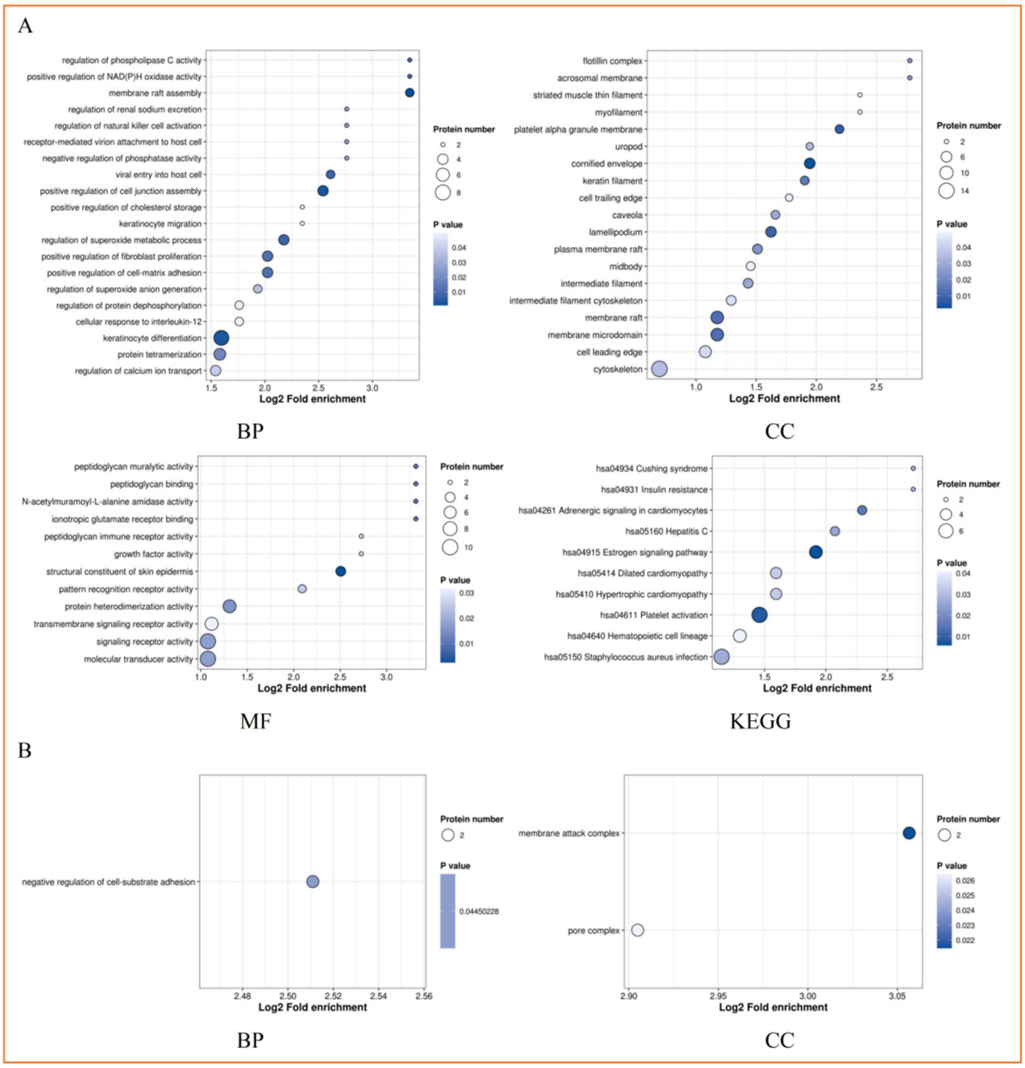

C

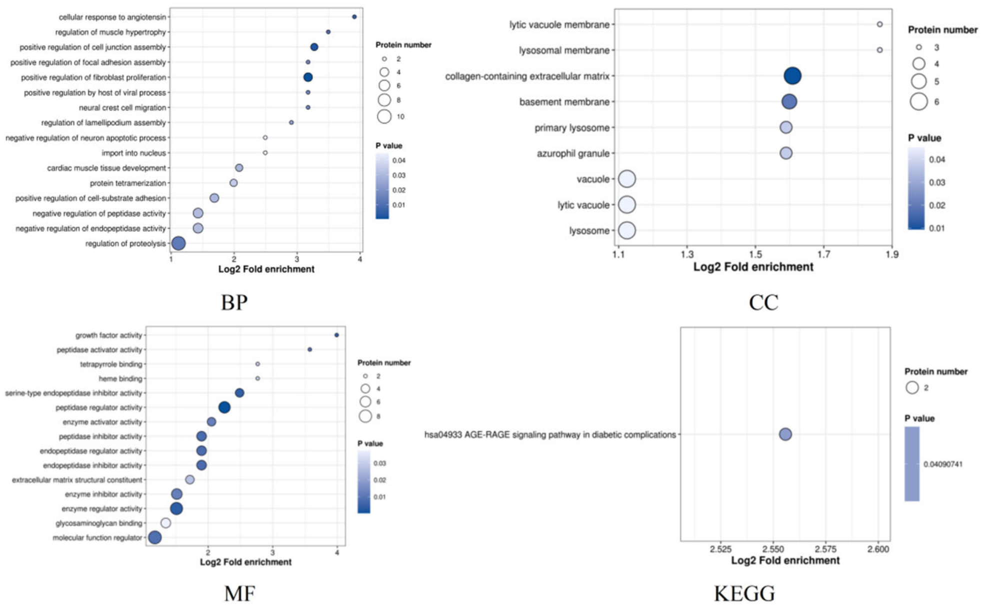
cellular response to angiotensin
regulation of muscle hypertrophy
positive regulation of cell junction assembly
positive regulation of focal adhesion assembly
positive regulation of fibroblast proliferation
positive regulation by host of viral process
neural crest cell migration
regulation of lamellipodium assembly
negative regulation of neuron apoptotic process
import into nucleus
cardiac muscle tissue development
protein tetramerization
positive regulation of cell-substrate adhesion
negative regulation of peptidase activity
negative regulation of endopeptidase activity
regulation of proteolysis
Log2 Fold enrichment
Protein number
P value
BP
lytic vacuole membrane
lysosomal membrane
collagen-containing extracellular matrix
basement membrane
primary lysosome
azurophil granule
vacuole
lytic vacuole
lysosome
Log2 Fold enrichment
Protein number
P value
CC
growth factor activity
peptidase activator activity
tetrapyrrole binding
heme binding
serine-type endopeptidase inhibitor activity
peptidase regulator activity
enzyme activator activity
peptidase inhibitor activity
endopeptidase regulator activity
endopeptidase inhibitor activity
extracellular matrix structural constituent
enzyme inhibitor activity
enzyme regulator activity
glycosaminoglycan binding
molecular function regulator
Log2 Fold enrichment
Protein number
P value
MF
hsa04933 AGE-RAGE signaling pathway in diabetic complications
Log2 Fold enrichment
Protein number
P value
0.04090741
KEGG

D

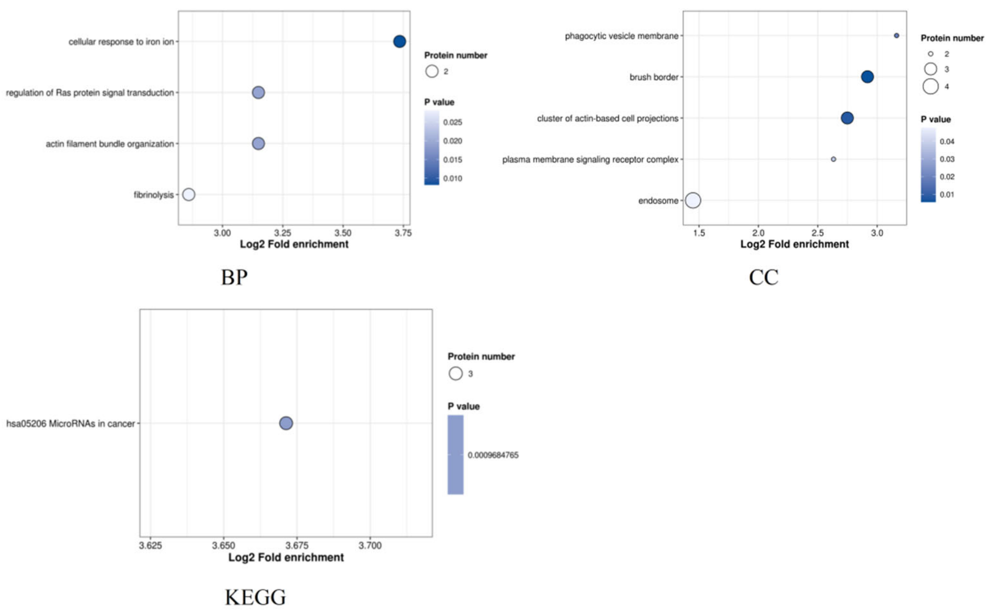
cellular response to iron ion
regulation of Ras protein signal transduction
actin filament bundle organization
fibrinolysis
Log2 Fold enrichment
Protein number
P value
BP
phagocytic vesicle membrane
brush border
cluster of actin-based cell projections
plasma membrane signaling receptor complex
endosome
Log2 Fold enrichment
Protein number
P value
CC
hsa05206 MicroRNAs in cancer
Log2 Fold enrichment
Protein number
P value
0.0009684765
KEGG

Supplemental Figure S4

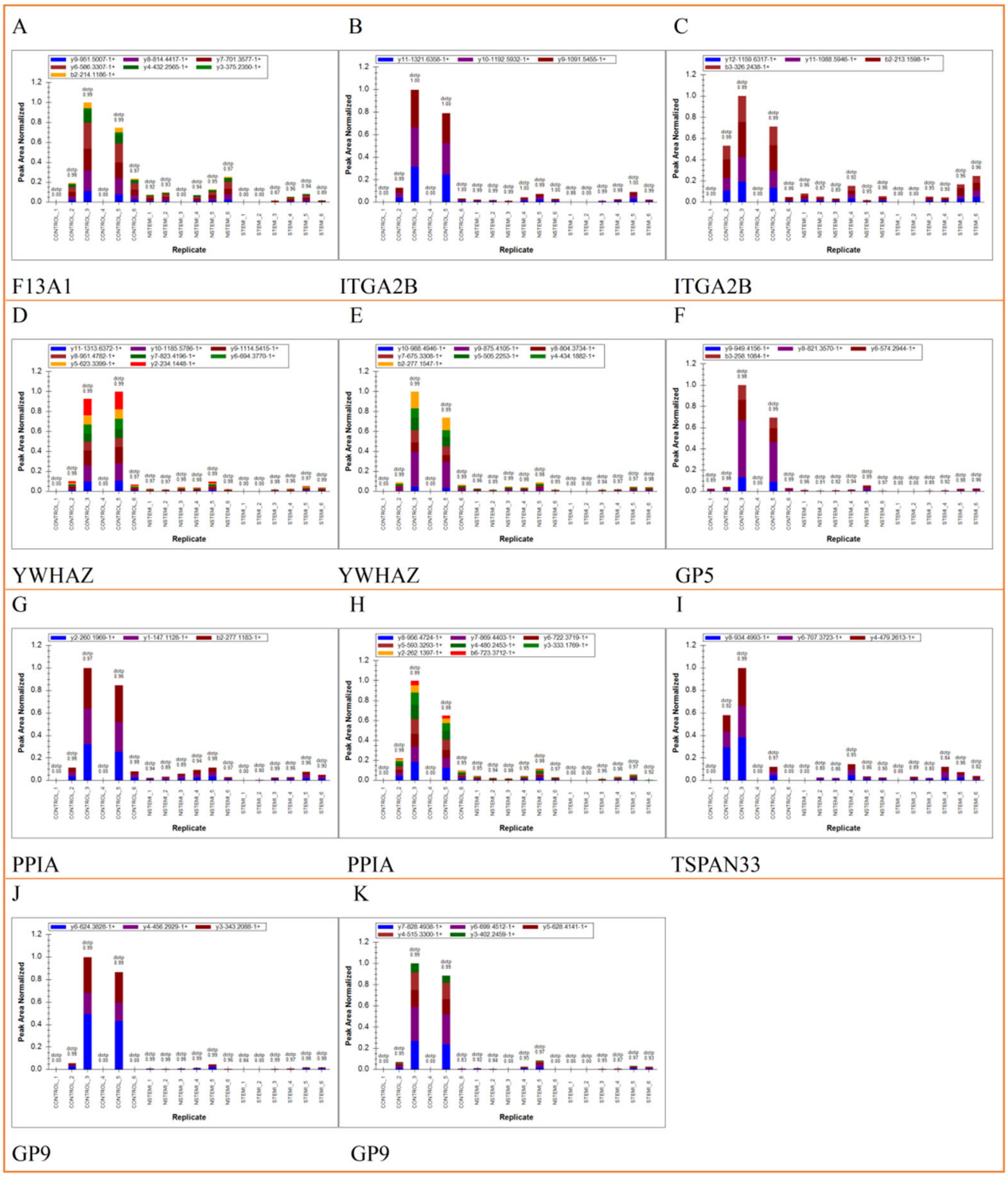

Supplemental Table S1 Detailed inclusion and exclusion criteria of the study.

Sample inclusion criteria			
**STEMI**	**NSTEMI**	**UA**	**CONTROL**
(1) The main diagnosis of discharge: STEMI;<br>(2) Age: 50-70 years old;<br>(3) onset time*: 2-8 hours;<br>(4) The test results of five cardiopulmonary indexes** have been abnormal.	(1) the main diagnosis of discharge: NSTEMI;<br>(2) Age: 50-70 years old;<br>(3) onset time*: 2-8 hours;<br>(4) The results of five five cardiopulmonary indexes** have been abnormal.	(1) The main diagnosis of discharge: UA;<br>(2) Age: 50-70 years old.	(1) Negative coronary angiography;<br>(2) Age: 50-70 years old.
**Sample exclusion criteria**			
(1) samples that do not meet the inclusion conditions;<br>(2) For patients with STEMI and NSTEMI, it is not the first time to get sick;<br>(3) For the CONTROL subjects, the required clinical data is incomplete (see Table 1 for details);<br>(4) Patients with serious diseases of heart, lung, liver, brain and kidney;<br>(5) Patients with autoimmune diseases;<br>(6) Patients with sexually transmitted diseases such as AIDS, syphilis and gonorrhea;<br>(7) Patients with tumors.			

* there are chest tightness, chest pain and other related symptoms, from symptoms to emergency diagnosis time is between 2-8 hours.

**Myo, CKMB, hs-cTnI, BNP, D-Dimer, see the notes in Table 1.

Supplemental Table S2 Multiple hypothesis test of factors with significant differences.

	group I	group J	p-adjusted
**Cardiac Biomarkers**			
BNP	STEMI	NSTEMI	0.015
	STEMI	UA	1.000
	NSTEMI	UA	0.232
**Laboratory data**			
AST	CONTROL	STEMI	<0.001
	CONTROL	NSTEMI	0.044
	CONTROL	UA	1.000
	STEMI	NSTEMI	0.717
	STEMI	UA	0.056
	NSTEMI	UA	0.820
ALT/AST	CONTROL	STEMI	<0.001
	CONTROL	NSTEMI	0.004
	CONTROL	UA	0.824
	STEMI	NSTEMI	0.205
	STEMI	UA	0.001
	NSTEMI	UA	0.007
TP	CONTROL	STEMI	0.103
	CONTROL	NSTEMI	0.003
	CONTROL	UA	0.166
	STEMI	NSTEMI	0.526
	STEMI	UA	0.995
	NSTEMI	UA	0.849
**Length of stay**			
	CONTROL	STEMI	<0.001
	CONTROL	NSTEMI	0.004
	CONTROL	UA	0.010
	STEMI	NSTEMI	1.000
	STEMI	UA	1.000
	NSTEMI	UA	1.000

Supplemental Table S3 Summary of Top 3 GO function classification of exosomal DEPs.

Group		Up DEPs Top 3 function	DEPs Num.	Down DEPs Top 3 function	DEPs Num.
STEMI-CONTROL	BP	regulation of biological	14	cellular component organization or biogenesis	14
		organic substance metabolic	11	regulation of biological	14
		nitrogen compound metabolic	9	organic substance metabolic	11
	CC	intracellular anatomical structure	13	extracellular region	13
		organelle	12	intracellular anatomical structure	12
		cytoplasm	11	cytoplasm	12
	MF	protein binding	10	protein binding	12
		heterocyclic compound binding	4	protein-containing complex binding	8
		organic cyclic compound binding	4	catalytic activity,acting on a protein	4
NSTEMI-CONTROL	BP	regulation of biological	20	regulation of biological	34
		response to stress	16	cellular component organization or biogenesis	31
		organic substance metabolic	15	anatomical structure development	29
	CC	extracellular region	22	intracellular anatomical structure	35
		extracellular space	17	cytoplasm	35
		intracellular anatomical structure	11	organelle	31
	MF	protein binding	16	protein binding	30
		ion binding	6	protein-containing complex binding	13
		antigen binding	5	catalytic activity,acting on a protein	9
UA-CONTROL	BP	regulation of biological	11	regulation of biological	34
		establishment of localization	8	cellular component organization or biogenesis	31
		organic substance metabolic	8	cellular response to stimulus	27
	CC	extracellular region	9	intracellular anatomical structure	33

		intracellular anatomical structure	9	cytoplasm	33
		cytoplasm	7	organelle	31
		protein binding	8	protein binding	29
	MF	enzyme regulator activity	5	protein-containing complex binding	14
		hydrolase activity	4	ion binding	11
		regulation of biological	6	organic substance metabolic	8
	BP	primary metabolic	4	primary metabolic	8
		nitrogen compound metabolic	4	regulation of biological	7
		extracellular region	5	extracellular region	7
**STEMI-NSTEMI**	**CC**	cytoplasm	5	extracellular space	7
		membrane-enclosed lumen	5	membrane	4
		protein binding	4	protein binding	3
	**MF**	organic cyclic compound binding	2	lipid transporter activity	2
		heterocyclic compound binding	2	lipid binding	2
		regulation of biological	13	regulation of biological	9
	**BP**	cellular component organization or biogenesis	10	organic substance metabolic	7
		organic substance metabolic	8	regulation of cellular protein metabolic	7
		intracellular anatomical structure	10	extracellular region	10
**STEMI-UA**	**CC**	cytoplasm	10	extracellular space	8
		extracellular region	9	intracellular anatomical structure	8
		protein binding	8	protein binding	6
	**MF**	ion binding	4	enzyme regulator activity	6
		protein-containing complex binding	3	protein-containing complex binding	5
**NSTEMI-UA**	**BP**	organic substance metabolic	8	regulation of molecular function	3
		regulation of biological	8	response to stress	3

	nitrogen compound metabolic	7	regulation of biological	3
	extracellular region	7	intracellular anatomical structure	4
**CC**	organelle	7	cytoplasm	4
	intracellular anatomical structure	7	organelle	3
	protein binding	6	enzyme regulator activity	3
**MF**	antigen binding	2	protein binding	2
	ion binding	2	ion binding	2

Supplemental Table S4 Summary of the GO function and KEGG pathway enrichment analysis of exosomal DEPs.

Group		smallest P value function/pathway	most DEPs function/pathway	greatest enichment degree function/pathway
STEMI-CONTROL	BP	regulation of protein acetylation<br>regulation of histone modification<br>positive regulation of chromatin organization	positive regulation of fibroblast proliferation<br>regulation of cell cycle phase transtion	regulation of protein acetylation<br>regulation of histone modification<br>positive regulation of chromatin organization
	CC	platelet alpha granule	platelet alpha granule	flotillin complex
	MF	ionotropic glutamate receptor binding	protein heterodimerization activity<br>protease binding	ionotropic glutamate receptor binding
	KEGG	osteoclast differentiation (hsa04380)	pathways in cancer (hsa05200)	osteoclast differentiation (hsa04380)
NSTEMI-CONTROL	BP	negative regulation of RNA biosynthetic	positive regulation of cell migration	sterol import<br>peptidyl-serine phosphorylation<br>negative regulation of stem cell proliferation
	CC	caveola	supramolecular fiber<br>supramolecular ploymer<br>supramolecular complex<br>cytoskeleton	tetraspanin-enriched microdomain<br>perinuclear endoplasmic reticulum
	MF	immunoglobulin receptor binding	integrin binding	SH2 domain binding<br>IgA binding
	KEGG	platelet activation (hsa04611)	platelet activation (hsa04611)<br>focal adhesion (hsa04510)	osteoclast differentiation (hsa04380)
UA-CONTROL	BP	membrane raft assembly	keratinocyte differentiation	regulation of phospholipase C activity<br>positive regulation of NAD(P)H oxidase activity<br>membrane raft assembly
	CC	cornified envelope	cytoskeleton	flotillin complex

	**MF**	structural constituent of skin epidermis	signaling receptor activity<br>molecular transducer activity	acrosomal membrane<br>peptidoglycan muralytic activity<br>peptidoglycan binding<br>N-acetylmuramoyl-L-alanine amidase activity<br>ionotropic glutamate receptor binding
	**KEGG**	estrogen signaling pathway (hsa04915)	platelet activation (hsa04611)<br>staphylococcus aureus infection (hsa05150)	cushing syndrome (hsa04934)<br>insulin resrstance (hsa04931)
**STEMI-NSTEMI**	**BP**	negative regulation of cell-substrate adhesion		
	**CC**		membrane attack complex<br>pore complex	
	**MF**		not available	
	**KEGG**		not available	
**STEMI-UA**	**BP**	positive regulation of fibroblast profileration	regulation of proteolysis	cellular response to angiotensin
	**CC**	collagen-containing extracellular matrix	vacuole<br>lytic vacuole<br>lysosome	lytic vacuole membrane<br>lysosomal membrane
	**MF**	peptidase regulator activity	peptidase regulator activity<br>enzyme regulator activity<br>molecular function regulator	growth factor activity
	**KEGG**		AGE-RAGE signaling pathway in diabetic complications (hsa04933)	
**NSTEMI-UA**	**BP**	cellular response to iron ion	same	cellular response to iron ion
	**CC**	brush border	endosome	phagocytic vesicle membrane
	**MF**		not available	
	**KEGG**		microRNAs in cancer (hsa05206)	

Supplemental Table S5 Summary of the cluster number of GO function and KEGG pathways.

	BP		CC		MF		KEGG	
	up	dowm	up	dowm	up	dowm	up	dowm
STEMI-CONTROL	1	8	5	3	3	1	1	12
NSTEMI-CONTROL	3	10	5	24	7	9	9	10
UA-CONTROL	15	6	4	10	18	4	3	4
STEMI-NSTEMI	0	0	0	3	0	0	1	0
STEMI-UA	1	15	1	0	2	13	0	2
NSTEMI-UA	1	4	2	0	0	3	0	2
